# The pleural tuberculosis–associated microenvironment promotes HIV-1 persistence by impairing CD8^+^ T cell–mediated viral control

**DOI:** 10.1172/jci.insight.201268

**Published:** 2026-08-20

**Authors:** Samantha Cronin, Jennifer Simpson, Andrea Pereyra-Casanova, Yuchen Li, Josefina Marín-Rojas, Freja A. Warner van Dijk, Katie Fisher, Daniel J. Buffa, Hafsa Rana, Zoï Vahlas, Joaquina Barros, Mariano Maio, Thomas R. O’Neil, Kirstie M. Bertram, Eunok Lee, Najla Nasr, Andrew N. Harman, Gabriela Turk, Maria Florencia Quiroga, Anthony D. Kelleher, Christel Vérollet, Luciana Balboa, Sarah Palmer, Gabriel Duette

**Affiliations:** 1 The University of Sydney, Faculty of Medicine and Health, Sydney, New South Wales, Australia.; 2 The Westmead Institute for Medical Rese arch, Centre for Virus Research, Sydney, New South Wales, Australia.; 3 Université de Toulouse, CNRS, Institut de Pharmacologie et de Biologie Structurale (IPBS), Toulouse, France.; 4 Universidad de Buenos Aires, Facultad de Medicina, Departamento de Microbiología, Parasitología e Inmunología, Buenos Aires, Argentina.; 5 Consejo Nacional de Investigaciones Científicas y Técnicas (CONICET) – Universidad de Buenos Aires. Instituto de Investigaciones Biomédicas en Retrovirus y SIDA (INBIRS). Buenos Aires. Argentina.; 6 Kirby Institute, University of New South Wales, Sydney, New South Wales, Australia.; 7 International Research Project CNRS “MAC-TB/HIV,” Toulouse, France, and Buenos Aires, Argentina.

**Keywords:** AIDS/HIV, Immunology, Infectious disease, Tuberculosis

## Abstract

*Mycobacterium tuberculosis* (*Mtb*), the causative agent of tuberculosis (TB), is the most common coinfection in people living with HIV-1. This coinfection is associated with accelerated HIV-1 disease progression and reduced survival. However, the immunological and virological mechanisms driving this progression are not completely understood. To address this knowledge gap, using pleural effusion samples from people living with HIV-1 and TB, we investigated how the HIV-1 genetic landscape and the anti–HIV-1 immune response are impacted by a TB-associated microenvironment. Our results revealed an enrichment of genetically intact HIV-1 and impaired CD8^+^ T cell–mediated antiviral response at this site of HIV-1/*Mtb* coinfection. Moreover, efficient CD8^+^ T cell activation was inhibited by lipids present in the TB-associated pleural effusion. These findings indicate that this immune microenvironment induced by TB promotes the persistence of cells infected with replication-competent HIV-1 by creating a niche of reduced antiviral immune pressure, potentially contributing to the worsened clinical outcomes observed in people living with HIV-1 and TB.

## Abstract 2

We found that Tuberculosis negatively impacts the immune response to HIV, explaining how Tuberculosis contributes to worsened clinical outcomes for people living with HIV.

## Introduction

Despite considerable progress with antiretroviral therapy (ART) improving the health and life expectancy of people living with HIV-1 (PLWH), the persistence of CD4^+^ T cells infected with genetically intact and replication-competent HIV-1 is a major barrier to HIV-1 cure. Although the majority of proviruses in PLWH are genetically defective (1), even a small pool of replication-competent viruses is sufficient to drive viral rebound upon ART interruption or failure (2–4). A deeper understanding of the factors shaping the size and genetic features of persistent HIV-1–infected cell populations is essential for the development of effective curative strategies.

Although CD8^+^ T cells are critical for controlling HIV-1 during acute infection, they eventually acquire an exhausted phenotype, which undermines viral control (5, 6). In addition, tissue-resident CD8^+^ T cells exhibit reduced cytotoxic capacity, allowing the virus to persist within anatomical niches such as lymph nodes (7, 8). Thus, inefficient CD8^+^ T cell–mediated antiviral activity allows for the persistence of genetically intact HIV-1, which contributes to viral rebound when ART is interrupted.

*Mycobacterium tuberculosis* (*Mtb*), the causative agent of tuberculosis (TB), is the most common coinfecting pathogen among PLWH worldwide (9). HIV-1/*Mtb* coinfection has been described as a *syndemic*, with the concurrence of both pathogens exacerbating the clinical severity of each other (10, 11). People living with HIV-1 and TB (PLWH/TB) are 18 times more likely to develop active TB and face reduced survival compared with individuals with only one of these infections (12, 13). Consequently, TB is the leading cause of death among PLWH, accounting for 33% of AIDS-related deaths in 2023 (9, 13).

Despite its frequency and clinical severity, little is known about how TB influences the size and genetic composition of HIV-1–infected cell populations. Studies of blood-derived cells have yielded conflicting results, with some reporting increased HIV-1 DNA during TB coinfection and others reporting decreases (14–16). This inconsistency highlights the need to investigate the systemic and compartment-specific effects of HIV-1/TB concurrence. While *Mtb*-specific CD4^+^ and CD8^+^ T cell responses are known to be impaired during TB disease, whether TB also compromises HIV-1–specific CD8^+^ T cell responses, potentially allowing for the pool of HIV-1–infected CD4^+^ T cells to expand, remains unexplored (17).

Active TB primarily occurs in the lungs; however, the bacteria can spread to other anatomic sites, including the pleural space (18). Tuberculous pleural effusion (TB-PE), an accumulation of fluid in the pleural space, develops in approximately 30% of TB patients (18) in response to the presence of mycobacteria or mycobacterial antigen in the pleura, triggering local inflammation and leukocyte infiltration (19, 20). In PLWH/TB, PE is more common and exhibits higher HIV-1 titers compared with paired plasma (21–23). Therefore, TB-PE represents a physiologically relevant ex vivo model to study the microenvironment at the site of HIV-1/*Mtb* coinfection and its impact on HIV-1 persistence (24–27).

In this study, we genetically characterized HIV-1 from TB-PE and examined the impact of TB-PE on antiviral CD8^+^ T cell responses. Our findings suggest that the TB-associated microenvironment creates a niche of reduced anti–HIV-1 immune pressure, permissive to the persistence of replication-competent HIV-1, which may contribute to the worsened clinical outcomes observed in PLWH/TB.

## Results

### HIV-1 proviral genetic landscape in PLWH/TB.

Despite the clinical consequences of HIV-1/*Mtb* coinfection, the impact of active TB on the HIV-1 proviral genetic landscape within circulating HIV-1–infected cells remains unclear. To address this, we analyzed HIV-1 proviral sequences from peripheral blood mononuclear cell (PBMC) samples obtained from 5 PLWH and 5 individuals with HIV-1 and active TB (PLWH/TB) (Table 1). The duration of ART ranged from 1 to 4 months for most participants, except for one individual who had been on ART for 8 years and experienced treatment failure (Table 1). Plasma viral loads ranged from less than 50 to 1,180 copies/mL, with no significant differences observed between the 2 groups (PLWH median 285, IQR 66.5–973, vs. PLWH/TB median 823, IQR 128.5–1,132) (Figure 1A). Consistent with previous studies, CD4^+^ T cell counts were significantly lower in PLWH/TB than in PLWH (PLWH median 441, IQR 388.5–542.5, vs. PLWH/TB median 132, IQR 109.5–360.5; *P* = 0.007) (Figure 1A) (28, 29).

To characterize the HIV-1 proviral genetic landscape in both groups, we used the full-length individual proviral sequencing (FLIPS) assay (30), obtaining a total of 434 HIV-1 DNA sequences from the 10 participants. As previously described in PLWH, the majority of sequences were genetically defective (65.9%–100%), with internal deletions being the most frequent defect (47.1%–94.9%) (Figure 1B) (1). Genetically intact HIV-1 sequences were identified in 9 of the 10 participants (Figure 1B). The proportion of genetically intact proviruses was 18% in PLWH/TB and 12% in PLWH (Figure 1C). When infection frequency was calculated, we observed that in PLWH/TB the frequency of infected cells per million CD4^+^ T cells tended to be higher for total (PLWH median 48.30, IQR 26.73–145.7, vs. PLWH/TB median 303.2, IQR 90.21–701.4), genetically defective (PLWH median 38.83, IQR 19.03–96.61, vs. PLWH/TB median 165.4, IQR 72.77–434.1), and genetically intact (PLWH median 4.20, IQR 1.92–23.41, vs. PLWH/TB median 36.82, IQR 8.36–110.1) HIV-1 DNA sequences (Figure 1D). These results suggest that concurrent TB may increase the frequency of cells infected with HIV-1.

### The proportion of genetically intact HIV-1 provirus is significantly higher at the site of the coinfection.

Most studies on HIV-1 infection in PLWH/TB have been conducted using blood samples. However, we and others have shown that at the site of the coinfection the TB-associated microenvironment can influence HIV-1 pathogenesis (21–24, 26, 27). For instance, in people who developed tuberculous pleural effusion (TB-PE), the number of HIV-1 RNA copies was higher in this fluid when compared with matched plasma (Figure 2A and Supplemental Figure 1; supplemental material available online with this article; https://doi.org/10.1172/jci.insight.201268DS1) (21–23). This result led us to investigate the impact of the TB-associated microenvironment on the HIV-1 genetic landscape at this site of coinfection. To this end, we accessed PBMC and pleural effusion–derived mononuclear cell (PEMC) samples from 3 PLWH/TB and performed FLIPS. One of these participants was receiving ART at the time of sample collection, with undetectable plasma viral load, but a relatively high HIV-1 RNA copy number in the TB-PE (Figure 2A and Table 2). In contrast, 2 of these participants were untreated for HIV-1 at the time of sampling (Figure 2A and Table 2). Interestingly, the proportion of genetically intact HIV-1 DNA sequences in PEMCs was higher in comparison with PBMCs across all 3 participants (0%–25.8% PBMCs; 17.8%–61.7% PEMCs) (Figure 2B). In participant 122, who was virally suppressed on ART, no intact provirus was found in the 4 sequences obtained from the blood. However, 17.8% of the proviral sequences were genetically intact in cells from the pleural space. Importantly, when HIV-1 infection frequency was calculated, we observed a significantly higher frequency of cells harboring genetically intact HIV-1 provirus in TB-PE when compared with peripheral blood (PBMC median 3.10, IQR 0–7.36, vs. PEMC median 128.2, IQR 121–183.1; *P* = 0.03, unpaired, 2-tailed *t* test on log-transformed values) (Figure 2C). Our data suggest that the TB-associated microenvironment may promote the persistence of genetically intact HIV-1 at the site of the coinfection in PLWH/TB.

### Genomic HIV-1 RNA derived from TB-PE is not genetically distinct from that derived from plasma.

Given the increased frequency of genetically intact HIV-1 and elevated HIV-1 RNA levels in TB-PE compared with paired blood (Figure 2, A–C, and Supplemental Figure 1), we investigated whether the pleural space represents a “sanctuary” site for enhanced viral replication. If this compartment was isolated from the peripheral circulation, genetic divergence between virus in the TB-PE and virus in the plasma would be expected (23). To test this, we sequenced HIV-1 RNA from plasma and TB-PE samples obtained from 2 untreated PLWH/TB (donors 127 and 134) using our recently developed HIV-1 RNA-seq assay (31). Maximum-likelihood phylogenetic analysis of these HIV-1 RNA sequences, spanning the *integrase*–3′ long-terminal repeat (LTR) genomic region, revealed no compartmental clustering patterns between the HIV-1 RNA derived from the pleural space and that derived from the plasma (Figure 2D).

### Effector memory CD4^+^ T cells are preferentially infected by HIV-1 in TB-PE.

To determine what cell types are preferentially infected during active TB, we performed scRNA-seq on PEMCs from 2 PLWH/TB (donors 127 and 154). Since the cellular composition of TB-PE in PLWH/TB has not been well defined, we compared our dataset with publicly available scRNA-seq data of PEMCs from TB-PE taken from participants with *Mtb* mono-infection (Figure 3A) (32). Clustering patterns were similar across datasets, suggesting comparable PEMC phenotypes and supporting the reliability of our data (Figure 3A).

Cell type identification analysis of the two PLWH/TB showed a predominance of CD4^+^ and CD8^+^ T cells (Figure 3B), as described in previous studies (33, 34). Further characterization of the CD4^+^ T cells identified central, effector, and naive populations as well as several T helper subsets (Figure 3C).

To characterize HIV-1–infected cells derived from TB-PE, we identified cells expressing HIV-1 transcripts. We first amplified proviral HIV-1 DNA by FLIPS in both donors and used these sequences as a reference to map HIV-1 transcripts in our dataset. HIV-1 transcripts were more prevalent in effector memory CD4^+^ T cells when compared with central memory and naive subsets (Figure 3D) (35). Our analysis also revealed that HIV-1 transcripts were enriched within the Th17 subset, followed by follicular helper T, Th2, and Th1/17 cells, respectively (Figure 3D).

### The majority of TB-PE–derived CD8^+^ T cells do not express canonical markers of tissue residency and exhibit an activated/exhausted phenotype.

Our results showing an increase in the proportion of genetically intact HIV-1–infected cells in TB-PE (Figure 2) suggest reduced immune pressure at this site of coinfection. Since CD8^+^ T cells are critical for HIV-1 control, we investigated whether CD8^+^ T cells derived from TB-PE are phenotypically dysfunctional. It has been previously reported that CD8^+^ T cells in the airways of PLWH exhibit a tissue-resident phenotype, defined by the expression of CD103 (36), which is associated with reduced cytotoxic potential (37, 38). To determine whether TB-PE is enriched for tissue-resident CD8^+^ T cells, we analyzed the phenotype of PEMC-derived CD8^+^ T cells from 2 PLWH/TB (donors 127 and 122) using a 19-color flow cytometry panel. Integration of both datasets followed by unbiased clustering identified 9 distinct clusters (Figure 4, A–D). CD103 expression was restricted to clusters 4, 8, and 9, indicating that although a fraction of the TB-PE–derived CD8^+^ T cells were potentially tissue resident, they did not represent the majority (Figure 4, C and D). CD69, another marker associated with tissue residency, was highly expressed in cluster 8 (Figure 4, A and D). However, this cluster also displayed high levels of the activation markers CD38 and human leukocyte antigen DR isotype (HLA-DR), suggesting that in this context CD69 primarily reflects activation rather than tissue residency (Figure 4D). In addition to cluster 8, CD38 and HLA-DR coexpression was also observed across clusters 6 and 7, together with the exhaustion markers programmed cell death protein 1 (PD-1) and T cell immunoglobulin and ITIM domain (TIGIT) (Figure 4D). PD-1 and TIGIT were also detected in cluster 5. CD39, which is associated with a terminally exhausted phenotype (39), was coexpressed with CD38 and HLA-DR in cluster 7 (Figure 4D). These data suggest the presence of an expanded pool of chronically activated CD8^+^ T cells with an exhausted phenotype. Notably, CCR5 was widely expressed across most clusters, consistent with migration of effector cells from the blood into the inflamed pleural space (Figure 4, B and D) (33). Clusters 1, 2, and 4 were characterized by the expression of CCR7, the costimulatory molecules CD27 and CD28, and the homeostatic cytokine receptor CD127, indicating a less differentiated, circulating phenotype (Figure 4D).

Together, these data show that TB-PE–derived CD8^+^ T cells are phenotypically diverse and that the majority do not exhibit a tissue-resident phenotype. Moreover, we identified CD8^+^ T cells displaying features of chronic activation and exhaustion, consistent with an inflammatory microenvironment and suggesting impaired antiviral function at the site of coinfection.

### HIV-1–specific CD8^+^ T cells exhibit a functionally exhausted phenotype in TB-PE.

Our analysis suggested the presence of chronically activated and exhausted CD8^+^ T cells at the site of the coinfection. However, this analysis did not allow us to determine whether the CD8^+^ T cells exhibiting this phenotype were specific for HIV-1. To address this, we combined the scRNA-seq data from donors 127 and 154 (Figure 3A) with paired single-cell T cell receptor (TCR) sequencing data to identify and characterize virus-specific CD8^+^ T cells. Comparing the TCRs from both donors with data from publicly available TCR databases (40), we identified CD8^+^ T cell–derived TCRs with specificity for HIV-1, EBV, HBV, influenza A, SARS-CoV-2, and CMV (Figure 4, E–J). We then analyzed the transcriptomic profile of these cells and compared the cytokine and cytotoxic signatures across the CD8^+^ T cells with different specificities to assess effector functionality. HIV-1–specific CD8^+^ T cells showed a strong cytokine signature relative to CD8^+^ T cells of other specificities, mainly driven by the expression of *IFNG*, indicating recent contact with the antigen (Figure 4, E and F). However, their cytotoxic signature was considerably lower than that of other virus-specific CD8^+^ T cells (Figure 4G). We observed that HIV-1–specific cells exhibited relatively lower expression of the cytotoxic genes granzyme A (*GZMA*), granzyme K (*GZMK*), and perforin 1 (*PRF1*) (Figure 4H). To further understand this result, we also assessed the relative exhaustion signature of HIV-1–specific CD8^+^ T cells. Notably, HIV-1–specific CD8^+^ T cells exhibited an exhausted signature, driven by high expression levels of *TIGIT*, cytotoxic T lymphocyte–associated protein 4 (*CTLA4*), and *HAVCR2*, which encodes the T cell immunoglobulin and mucin domain containing-3 (TIM-3) protein (Figure 4J). Collectively, these results indicate that HIV-1–specific CD8^+^ T cells are phenotypically exhausted, potentially contributing to reduced immune pressure at the site of the coinfection.

### The TB-associated microenvironment inhibits effector CD8^+^ T cell functionality.

Exhaustion of CD8^+^ T cells is a well-established hallmark of HIV-1 pathogenesis (5, 6). Therefore, our finding that HIV-1–specific CD8^+^ T cells in TB-PE are functionally exhausted is not unique to coinfection with *Mtb*. To better understand the specific contribution of the TB-associated microenvironment to CD8^+^ T cell functionality, we developed an in vitro model using pooled acellular fractions of TB-PE from individuals with active pleural TB (Figure 5A) (27). In this model, we stimulated CD8^+^ T cells isolated from healthy donors with anti-CD3/CD28 antibodies to mimic antigenic stimulation. Stimulated and unstimulated CD8^+^ T cells were cultured in the presence or absence of TB-PE to determine whether the inflammatory TB-associated microenvironment affected the CD8^+^ T cell effector response (Figure 5A). We first performed bulk RNA-seq to characterize the transcriptomic profile of cells stimulated with anti-CD3/CD28 antibodies exposed to TB-PE. Differential gene expression analysis identified 228 upregulated genes and 237 downregulated genes between controls and cells exposed to TB-PE (Figure 5, B and C, and Supplemental Figure 2). Gene Ontology pathway analysis revealed that cell pathways and genes associated with T cell effector functions were significantly downmodulated by TB-PE (Figure 5, D–F). Moreover, cell pathways related to chemotaxis were upregulated, reflective of the inflammatory nature of the TB-associated microenvironment (Figure 5E). We further validated these results by measuring the cell-surface protein expression of the activation markers CD69, CD25, and HLA-DR. Consistent with the transcriptomic profile shaped by TB-PE, the presence of TB-PE impaired the upregulation of these activation markers upon T cell stimulation (Figure 5G). To confirm whether the TB-PE–mediated inhibition of T cell activation is a common feature of pleural TB, we tested additional pooled and individual TB-PE samples. In addition, to test that this immunosuppressive phenotype was specific to TB-PE rather than a feature of the pleural environment, we also included pleural effusion from individuals with heart failure (HF-PE). Unlike HF-PE, both the additional pool and the individual TB-PE samples reproduced the inhibitory effect (Figure 5H). These findings suggest that the impairment of CD8^+^ T cell activation is a consistent feature of TB-PE.

To further characterize the impact of the TB-associated microenvironment on effector functions, we also measured the expression of hypoxia-inducible factor 1α (HIF-1α), since this transcription factor is necessary for an efficient T cell effector response (41). Cells exposed to TB-PE exhibited reduced expression of HIF-1α (Figure 5I).

Taken together, these data indicate that the TB-associated microenvironment impairs CD8^+^ T cell activation and effector functionality upon antigenic stimulation.

### HIV-1–specific CD8^+^ T cell responses are inhibited by exposure to TB-PE.

Our results demonstrate that the TB-associated microenvironment impairs the effector functionality of CD8^+^ T cells after antigen stimulation. This prompted us to investigate whether TB-PE exposure can also inhibit the antiviral function and HIV-1–specific effector responses mediated by CD8^+^ T cells derived from PLWH. To address this question, we stimulated PLWH-derived PBMCs with a pool of HIV-1 peptides to expand HIV-1–specific CD8^+^ T cells for 14 days. After expansion, cells were restimulated with the HIV-1 peptides in the presence or absence of TB-PE, to induce the expression of the degranulation markers CD107a and CD107b and the effector cytokines IFN-γ and TNF-α (Figure 6A). Importantly, cells treated with TB-PE exhibited significantly lower expression of CD107a/b, IFN-γ, and TNF-α when compared with cells expanded without TB-PE (Figure 6, B and C). Notably, we observed no significant differences between stimulated cells and cells stimulated in the presence of HF-PE (Figure 6, B and C). These results were further confirmed when cells from healthy donors were stimulated with a pool of peptides for common viruses (CMV, EBV, and influenza; CEF peptide pool), in the presence or absence of TB-PE (Supplemental Figure 3). Overall, these data demonstrate that the TB-associated microenvironment inhibits the ability of CD8^+^ T cells to mount an efficient antiviral effector response.

### TB-PE prevents HIV-1–specific CD8^+^ T cells from acquiring cytotoxic function.

Considering that TB-PE inhibits efficient TCR-dependent T cell activation, we hypothesized that the TB-associated microenvironment could also impair the differentiation of CD8^+^ T cells into a cytotoxic phenotype upon antigen encounter, leading to inefficient killing of HIV-1–infected CD4^+^ T cells. To assess this, PBMCs from PLWH were expanded with HIV-1 peptides in the presence or absence of TB-PE for 14 days. After expansion, CD8^+^ T cells were cocultured in the absence of TB-PE with autologous CD4^+^ T cells superinfected with HIV-1 in vitro (Figure 7A). Notably, at the time of coculture, the number of CD8^+^ T cells was normalized based on the expression of CD107a/b, ensuring that equal numbers of cytotoxic CD8^+^ T cells were included across conditions (Figure 7A). As expected, HIV-1–specific CD8^+^ T cells expanded with peptides alone significantly reduced the number of HIV-1–infected CD4^+^ T cells when compared with the control culture of CD4^+^ T cells (Figure 7B). In contrast, HIV-1–specific CD8^+^ T cells expanded in the presence of TB-PE were unable to eliminate HIV-1–infected CD4^+^ T cells when compared with CD8^+^ T cells expanded with peptides alone (Figure 7B). This finding was further confirmed when cells from healthy donors were expanded with the CEF peptide pool in the presence or absence of TB-PE and cocultured with target CD4^+^ T cells pulsed with CEF peptides (Supplemental Figure 4). These results indicate that the TB-associated microenvironment hampers the differentiation of HIV-1–specific CD8^+^ T cells into fully cytotoxic effectors upon antigen exposure.

### CD8^+^ T cells exposed to TB-PE accumulate neutral lipids.

To explore the mechanisms underlying the immunosuppressive effect of TB-PE on CD8^+^ T cells, we leveraged our bulk RNA-seq dataset generated using our in vitro model (Figure 5, B and C, and Supplemental Figure 2). Since stimulation with anti-CD3/CD28 antibodies could potentially mask direct effects of TB-PE on exposed cells, we first focused our analysis on cells treated with TB-PE in the absence of activating stimuli. Interestingly, the 2 most upregulated genes were fatty acid–binding protein 4 (*FABP4*) and lipoprotein lipase (*LPL*) (Supplemental Figure 2), which are involved in fatty acid trafficking and storage. This result prompted us to further characterize cellular pathways associated with fatty acid transport and storage. In both unstimulated and anti-CD3/CD28–stimulated conditions, TB-PE exposure resulted in the upregulation of pathways associated with lipid transport and storage (Figure 8, A and C). Consistently, TB-PE–exposed cells showed increased expression of genes involved in lipid transport and metabolism, including *LPL*, ATP-binding cassette transporter A1 (*ABCA1*), and carnitine palmitoyltransferase 1A (*CPT1A*), irrespective of anti-CD3/CD28 stimulation (Figure 8, B and D).

To assess whether TB-PE exposure promotes intracellular lipid accumulation, we stained CD8^+^ T cells with BODIPY 493/503, a fluorescent dye that labels neutral lipids and can be used to visualize and quantify intracellular lipid accumulation by fluorescence microscopy. CD8^+^ T cells exposed to TB-PE for 24 hours exhibited higher levels of neutral lipids than unexposed controls, as shown by increased mean fluorescence intensity in TB-PE–exposed cells (*P* < 0.0001) (Figure 8, E–G).

Together, these results show that TB-PE exposure induces a transcriptional program associated with lipid transport and storage and promotes intracellular accumulation of neutral lipids in CD8^+^ T cells.

### TB-PE exposure downmodulates oxidative phosphorylation and glycolysis.

Efficient T cell responses require increased glycolysis and oxidative phosphorylation (OXPHOS) (42). It has recently been reported that lipid-enriched tumor microenvironments can promote lipid uptake and storage in infiltrating CD8^+^ T cells, leading to the downmodulation of glycolysis and OXPHOS and, ultimately, impaired effector function (43). To determine whether TB-PE exposure affects activation-induced glycolysis and OXPHOS, we assessed the metabolic profile of CD8^+^ T cells exposed to TB-PE using a Seahorse cell flux analyzer. Oxygen consumption rate and proton efflux rate were measured as indicators of OXPHOS and glycolysis, respectively (Figure 8H). The intracellular ATP production rate derived from glycolysis (glycoATP) or OXPHOS (mitoATP) was also quantified (Figure 8H). Both OXPHOS and glycolysis were significantly reduced in TB-PE–treated cells compared with controls (Figure 8H). These results suggest that TB-PE exposure impairs the metabolic reprogramming required for CD8^+^ T cell activation, potentially contributing to reduced effector function.

### TB-PE–derived lipids inhibit CD8^+^ T cell activation.

Finally, to determine whether lipids derived from TB-PE can directly affect CD8^+^ T cell effector functions, we separated TB-PE into 2 fractions: one enriched in polar metabolites (PF-PE) and another comprising lipids (Lip-PE). As a proxy for T cell effector function, we measured the expression of the activation markers CD69 and CD25 following stimulation with anti-CD3/CD28 antibodies. CD8^+^ T cells were preincubated with PF-PE or Lip-PE for 1 hour before stimulation with anti-CD3/CD28 antibodies. After 24 hours, CD8^+^ T cells exposed to Lip-PE, but not those exposed to PF-PE, exhibited significantly reduced expression of both CD69 and CD25 (Figure 8I). These results suggest that lipids present in the TB-associated microenvironment contribute to impaired CD8^+^ T cell activation.

In summary, our results suggest that the higher proportion of genetically intact HIV-1–infected cells observed at the site of coinfection may result, at least in part, from impaired CD8^+^ T cell–mediated control of HIV-1. This impairment is associated with reduced effector differentiation and function, potentially driven by lipids present in the TB-associated microenvironment.

## Discussion

While extensive clinical evidence highlights the synergistic interaction between HIV-1 and *Mtb*, leading to worsened outcomes for PLWH/TB, little is known about how TB disease can promote HIV-1 persistence. In this study, we addressed this gap by characterizing the genetic HIV-1 proviral landscape in PLWH/TB and the impact of a TB-associated microenvironment on CD8^+^ T cell–mediated HIV-1 control. Our findings provide two key insights: (a) TB-PE is enriched in genetically intact HIV-1, and (b) suppresses effective HIV-1–specific CD8^+^ T cell responses. We propose that the reduced immune pressure on HIV-1 infection at the site of HIV-1/*Mtb* coinfection creates a permissive niche for the persistence of genetically intact and replication-competent virus.

A recent study showed that circulating CD4^+^ T cells from PLWH with a history of active pulmonary TB harbored higher levels of intact provirus in comparison with those without a history of active TB (14). In line with these findings, we observed in our cohort that both defective and intact HIV-1 infection frequencies tended to be higher in PLWH/TB than in PLWH without TB. Although these differences did not reach statistical significance, likely because of the small sample size, they are consistent with prior reports showing elevated levels of HIV-1 DNA in PLWH/TB (14, 16). Importantly, these observations align independently of cohort characteristics and regardless of treatment history for HIV-1 or TB.

Despite increasing research on HIV-1/*Mtb* coinfection, characterization of the anatomic sites of coinfection remains challenging. Although research in nonhuman primates provided important insights into pathogen dynamics, these studies mainly assess the impact of HIV-1 infection on TB outcomes and do not fully recapitulate the pathology observed in humans (44–46). Thus, models using human samples are essential to better understand coinfection in PLWH/TB. In this study, we used TB-PE as a model of a site of coinfection to characterize an inflammatory environment shaped by *Mtb*. Consistent with previous reports, we observed higher HIV-1 RNA levels in pleural fluid compared with plasma (21, 23). Whether this increase reflects an enrichment of cells carrying genetically intact and replication-competent virus at the site of the coinfection has not been investigated. Therefore, using near-full-length sequencing assays, we genetically characterized HIV-1 proviruses in the pleural compartment. Our findings reveal a higher frequency of cells harboring genetically intact proviruses in TB-PE. This enrichment of genetically intact HIV-1 could be explained by two potential mechanisms: (a) enhanced local viral replication promoted by the inflammatory TB microenvironment and/or (b) reduced antiviral immune pressure within this environment. Given the high mutation rate of HIV-1, an isolated compartment would be expected to display genetic divergence between viruses. However, our phylogenetic analysis of paired plasma and TB-PE samples did not reveal genetic divergence, suggesting that the elevated levels of HIV-1–infected cells at the coinfection site in these individuals cannot be attributed to an isolated “sanctuary” supporting independent viral replication. Previous reports sequencing the HIV-1 envelope region did identify genetic differences between PE and plasma but also demonstrated viral trafficking between the two compartments (23).

Substantial efforts in the HIV-1 field have aimed to identify CD4^+^ T cell subsets preferentially infected by HIV-1. Consistent with previous studies in PLWH, we found that TB-PE–derived effector memory CD4^+^ T cells were enriched in HIV-1 transcripts (47–49). Interestingly, infected cells were primarily concentrated within the Th17, follicular helper T cell, Th2, and Th1/17 subsets. This result contrasts with previous reports showing that blood-derived Th1 CD4^+^ T cells are enriched for HIV-1 RNA in PLWH but aligns with recent studies demonstrating preferential infection of Th17 CD4^+^ T cells in tissue (47, 49–54). Our findings suggest that this TB-associated microenvironment may promote the accumulation of preferential targets for HIV-1 infection at the site of the coinfection.

To determine whether the observed enrichment of genetically intact HIV-1 at the HIV-1/*Mtb* coinfection site results from reduced immune pressure, we assessed CD8^+^ T cell–mediated control of HIV-1 within this compartment. Recent evidence indicates that tissue-resident CD8^+^ T cells display reduced cytotoxic capacity, contributing to HIV-1 persistence in tissues such as lymph nodes (7, 8). In contrast to immunological studies of bronchoalveolar lavage from PLWH, we observed that only a fraction of the CD8^+^ T cell population in TB-PE exhibited a tissue-resident phenotype (37, 55). These differences may reflect the distinct cellular composition of the lung parenchyma versus the exudative nature of pleural fluid. Importantly, HIV-1–specific CD8^+^ T cells in TB-PE exhibited an exhausted phenotype with diminished cytotoxic potential. Because CD8^+^ T cell exhaustion is a well-known feature of HIV-1 infection, independent of *Mtb* coinfection (56), we sought to define the specific impact of the TB-associated microenvironment. Using our in vitro model, we demonstrated that the inflammatory pleural environment impairs CD8^+^ T cell effector functionality, leading to suboptimal elimination of HIV-1–infected cells. Interestingly, although TB-PE is enriched in pro- and antiinflammatory cytokines, our results indicate that TB-PE–derived lipids are the key mediators of CD8^+^ T cell dysfunction within this microenvironment. Previous characterization from our group suggests that these lipids are likely host-derived rather than from the mycobacteria (57). Increasing evidence supports a role for lipid-mediated immunoregulation of CD8^+^ T cells, particularly within the tumor microenvironment (58–60), where lipid accumulation has been associated with impaired glycolysis and OXPHOS, both of which are required for effective effector function (43).

Taken together, our findings support the idea that the higher proportion of cells infected with genetically intact HIV-1 in PLWH/TB may arise from reduced immune pressure. Although pleural TB is a transient condition, the establishment of a pool of cells enriched in genetically intact proviruses may have long-term implications for the persistence of the HIV-1 reservoir, underscoring the importance of understanding immune functionality at the site of HIV-1/*Mtb* coinfection.

Several limitations should be acknowledged in this study. First, access to these rare samples was limited, and our genetic comparison between proviruses derived from blood and pleural fluid included only 3 PLWH/TB. Despite the small sample size, our findings were consistent, showing a high proportion of genetically intact HIV-1 in this compartment. Second, our single-cell transcriptomic analysis was performed in only 2 participants, and matched blood samples were unavailable. Nevertheless, comparison with publicly available datasets revealed similar clustering patterns, supporting the validity of our observations. Additionally, to determine the specificity of CD8^+^ T cell–derived TCRs, we relied on public databases. While these repositories are inherently limited to reported TCR sequences, this limitation is comparable to studies using tetramers, in which analyses are restricted to predefined specificities. Finally, TB-PE represents a specific TB-associated microenvironment and may not fully reflect other tissue sites, such as granulomas. Whether other respiratory infections induce similar effects also remains to be determined. Additionally, owing to limited availability of HF-PE, non-TB PE controls were not added to every experiment. Nevertheless, TB-PE provides a physiologically relevant human model that helps overcome key limitations in the field, including restricted access to human tissue compartments.

Our work provides new insights into the effect of a TB-associated microenvironment and the immune mechanisms that may contribute to worsened virological outcomes in PLWH/TB. It underscores the need for further exploration of therapeutic interventions aimed at minimizing the impact of active TB on the HIV-1 reservoir. These findings also have important implications for the HIV-1 cure field, as we provide evidence that, at the site of coinfection, impaired HIV-1–specific CD8^+^ T cell responses may contribute to the accumulation of genetically intact proviruses in PLWH/TB, representing an additional challenge for cure-focused interventions in this population.

## Methods

### Sex as a biological variable

Both male and female individuals were included in the study, but because of the limited number of female participants, sex-based analyses could not be reliably performed.

### Participant cohort and clinical samples

To genetically characterize HIV-1 proviral sequences, PBMCs isolated by Ficoll density gradient centrifugation were collected from 8 PLWH/TB and 5 PLWH and cryopreserved at –80°C. Samples obtained from PLWH/TB were collected at the Hospital F.J. Muñiz and the Hospital Fernández, Buenos Aires, Argentina. Samples derived from PLWH were collected through the Grupo Argentino de Seroconversión study group (61). The diagnosis of active TB was bacteriologically confirmed by either positive acid-fast bacilli smear, Ziehl-Nielsen smear, or *Mtb* culture. Relevant clinical data of each participant are described in Tables 1 and 2. Four of the PLWH/TB had been diagnosed with pleural TB disease requiring therapeutic thoracentesis at the Hospital F.J. Muñiz in Buenos Aires, Argentina. Therapeutically aspirated TB-PEs were collected as described below and stored at –80°C until use.

To investigate the effect of TB-PE on HIV-1–specific CD8^+^ T cell responses, PBMCs were obtained from 5 PLWH on ART recruited through the Primary HIV and Early Disease Research: Australian Cohort (PHAEDRA) / CORE01 Acute Infection and Early Disease Research Program (AIEDRP) prospective observational protocol in Australia, which enrolled individuals during acute or early HIV-1 infection (62). Relevant clinical data of each participant are described in Supplemental Table 2.

### Pleural effusion samples

Pleural effusions (PEs) were obtained by therapeutic thoracentesis at the Hospital F.J. Muñiz from 3 PLWH/TB (Table 2) and 13 HIV-1–negative people with PE (Supplemental Table 1). The diagnosis of TB pleurisy was based on a positive Ziehl-Nielsen stain or Lowestein-Jensen culture from PE and/or histopathology of pleural biopsy, further supported by an *Mtb*-induced IFN-γ response and an adenosine deaminase–positive test. Exclusion criteria included the presence of concurrent communicable or non-communicable (cancer, diabetes, or steroid therapy) conditions, and multi-drug-resistant *Mtb*. For the HIV-1–negative donors, exclusion criteria also included a positive HIV-1 test. PE were collected and cryopreserved as detailed previously (27, 57).

### Pleural effusion pools

PE from recruited HIV-1–negative participants were divided into 2 pools depending on etiology. The first pool was composed of samples from 9 participants diagnosed with TB-PE, and the second was composed of 4 patients with heart failure–induced transudates (HF-PE). TB-PE samples were classified as exudates according to ref. 63. Pools were prepared as previously described (27, 57).

For PE-treated experimental conditions, 20% vol/vol PE was added to the cell culture, as previously optimized by our group (26, 57, 64, 65).

### Primary CD8+ T cell isolation from healthy donors

PBMCs were obtained by Ficoll-Hypaque (Sigma-Aldrich) density gradient centrifugation from buffy coats of healthy donors. CD8^+^ T cells were isolated from PBMCs by negative selection (catalog 130-096-495, Miltenyi Biotec).

### Full-length individual proviral sequencing

Near-full-length HIV-1 proviral sequences were obtained using the full-length individual proviral sequencing (FLIPS) assay as previously described (30, 66).

### Identification of genetically intact and defective HIV-1 proviruses

Proviruses were considered intact if they passed the criteria of the Proviral Sequence Annotation and Intactness Test (https://psd.cancer.gov/tools/tool_index.php) and met the following criteria: (a) all open reading frames (ORFs) had an intact start codon, (b) regulatory/accessory protein ORFs were intact (no insertions or deletions >5% of the ORF length, stop codons, or frameshift mutations), and (c) sequences possessed either an intact major splice donor (MSD) or cryptic MSD 4 nucleotides downstream of MSD 1 in the event MSD 1 was mutated. Defective proviruses were categorized as previously described (67–69).

### Plasma-derived RNA using long-range sequencing assay

Genomic HIV-1 RNA derived from the plasma and PE was sequenced with the plasma-derived RNA using long-range sequencing (PRLS) assay as previously described (31). This assay enables sequencing of individual HIV-1 RNA genomes spanning either *gag* to the 3′-LTR (*gag*–3′; ~8.3 kb) or, where low viral load results in low efficiency, *int* to the 3′-LTR (*int*–3′; ~5 kb). For the plasma sample of donor 127, we applied the *gag*–3′ assay. For all other samples, the *int*–3′ assay was used.

### Phylogenetic analysis

Genomic HIV-1 RNA sequences were trimmed to the *integrase*–3′-LTR region for phylogenetic analysis between sequences derived from the plasma and the PE. Maximum-likelihood trees were constructed for matched plasma- and PE-derived sequences. Sequences with an internal deletion (>100 bp) or those defined as hypermutant by the Los Alamos National Laboratory Hypermut tool (https://www.hiv.lanl.gov/content/sequence/HYPERMUT/hypermutv3.html) were excluded. Sequences were visualized using maximum-likelihood phylogenetic trees constructed with FastTree version 2.2.0 (70) using the generalized time-reversible model. Branch support values were calculated using 1,000 bootstrap replicates, and resulting phylogenetic trees were visualized and annotated using ggtree (71).

### High-parameter flow cytometry

PEMCs’ immunophenotype was determined using a 19-color flow cytometry panel (72). Details of the antibodies specific for CD28, CD8, HLA-DQ, CD161, CD154, CD4, CD39, CCR7, HLA-DR, PD-1, CD27, CCR5, CD3, CD69, TIGIT, CD103, CD127, and CD38 are listed in Supplemental Table 3. A total of 2.5 × 10^6^ cells per donor were stained as previously reported (72).

Data were analyzed using uniform manifold approximation and projection (UMAP) for dimensionality reduction and FlowSOM for clustering within the Spectre R package (73, 74).

### Single-cell RNA and T cell receptor sequencing library

Single-cell suspensions of PEMCs were prepared for the BD Rhapsody single-cell sequencing pipeline according to the manufacturer’s instructions. Twelve thousand PEMCs were processed using the BD Rhapsody system, and RNA and TCR libraries were generated using the TCR/BCR Full Length, mRNA Whole Transcriptome Analysis (WTA), and Sample Tag kits (BD Biosciences). Libraries were sequenced at 20,000 reads per cell for WTA and TCR libraries using the NovaSeq platform (Illumina).

### Single-cell RNA and T cell receptor sequencing analysis

Initial data analysis used the BD Seven Bridges desktop app and standard pipelines. Further analysis was performed using the R packages Seurat (75) and scRepertoire (76) for differential gene expression analysis and TCR repertoire identification, respectively. Pathway analysis was conducted with the R package Escape (77). Cell type identification was performed using the SingleR and CellDex (78) packages, with the associated reference libraries including the Monaco (79) reference for CD8^+^ T cell subset identification, the Novershtern (80) reference to identify central and effector memory cell subsets, and the Database of Immune Cell Expression (DICE; https://dice-database.org/#) (81) reference to identify CD4^+^ T cell subsets. HIV-1–infected cells were identified using the HIV-1 consensus sequences previously obtained through the FLIPS assay for each participant as a reference to identify HIV-1 transcripts via the Seven Bridges pipeline. TCR sequences for cells specific for HIV-1, CMV, EBV, influenza, and SARS-Cov2 were obtained from the VDJdb database (40). Single-cell RNA-seq data from PEMCs from participants with pleural TB alone were obtained from publicly available datasets (accession numbers HRA000910 and HRA00036, Genome Sequence Archive in the BIG Data Center, Beijing Institute of Genomics, Chinese Academy of Sciences) and used for comparative analysis.

### Bulk RNA-seq library

Isolated CD8^+^ T cells from healthy donors were activated using anti-CD3/CD28 antibodies (Miltenyi Biotec) in the presence or absence of TB-PE. Resting cells were also cultured in the presence or absence of TB-PE. After 20 hours of stimulation, cells were collected to extract RNA, and library preparation was performed as previously described (27).

### Bulk RNA-seq analysis

RNA-seq read quality control and processing, differential expression analysis, and pathway enrichment analysis were performed as described in previous work (27).

### Glycolysis and oxidative phosphorylation rate measurement

Purified CD8^+^ T cells were stimulated with anti-CD3/CD28 antibodies (Miltenyi Biotec) in the presence or absence of TB-PE. After 24 hours, cells were cultured in RPMI-based Seahorse medium (Agilent) supplemented with 10 mM d-glucose, 1 mM pyruvate, and 2 mM glutamine and plated in a Seahorse 24-well plate at a concentration of 7 × 10^5^ cells per well. Proton efflux rate and oxygen consumption rate were measured using a Seahorse XFe24 analyzer. ATP production rates were measured using the Seahorse XF Real Time ATP Rate Assay kit as described previously (27).

### Preparation of viral stocks and HIV-1 infection

HIV-1^NL4-3^ stocks were produced by transfection of HEK293T cells (American Type Culture Collection), and CD4^+^ T cells were infected as previously detailed (27, 35).

### CD8+ T cell–mediated cytotoxicity assay

#### Expansion of CD8^+^ T cells (effector cells).

The expansion of virus-specific CD8^+^ T cells was performed as previously described (82). Cryopreserved PBMCs from PLWH or healthy donors were thawed and rested overnight in RF10 (RPMI-1640 [Gibco] containing 10% fetal bovine serum [FBS; Gibco], 1× GlutaMAX [Gibco], 100 U/mL penicillin, and 100 µg/mL streptomycin [Gibco]) supplemented with 100 U/mL IL-2 (Miltenyi Biotec). The following day, cells were cultured in AIM-V medium (Thermo Fisher Scientific) in the presence or absence of TB-PE for 30 minutes. After preincubation, PBMCs from PLWH were stimulated with 4 μg/mL Gag, Pol, and Nef peptide pools (HIV-1 peptide pool; NIH AIDS Reagent Program, Division of AIDS, NIAID), while cells from healthy donors were treated with 4 μg/mL of a peptide pool derived from CMV, EBV, and influenza (CEF peptide pool; NIH AIDS Reagent Program) for 2 hours at 37°C.

Cells were washed with RF10 and cultured in RF10 supplemented with 100 U/mL IL-2 (Miltenyi Biotec) and 10% vol/vol TB-PE for 12 days. Culture medium was replaced every 48–72 hours. Expanded CD8^+^ T cells were isolated by negative selection (catalog 130-096-495, Miltenyi Biotec).

The frequency of antigen-specific and cytotoxic CD8^+^ T cells was determined by CD107a/b staining following peptide restimulation, as previously described (82). The proportion of CD107a/b^+^ cytotoxic CD8^+^ T cells was used to normalize effector cell numbers in subsequent coculture assays, as described below.

#### Expansion of CD4^+^ T cells (target cells).

CD4^+^ T cells were expanded from cryopreserved PBMCs from PLWH or healthy donors. Cells were cultured in RF10 medium supplemented with 100 U/mL IL-2 and 0.5 μg/mL anti-CD3/CD8 bispecific antibody (ARP-12277, obtained from Johnson Wong and Galit Alter through the NIH AIDS Reagent Program).

PBMCs from PLWH were cultured in the presence of antiretrovirals (100 nM efavirenz, 100 ng/mL enfuvirtide, and 30 μM raltegravir; MilliporeSigma) to prevent viral spread and reinfection. Treatment with the anti-CD3/CD8 bispecific antibody selectively depleted CD8^+^ T cells, enriching for activated CD4^+^ T cells. On day 11, CD4^+^ T cells were purified by negative selection (catalog 130-096-533, Miltenyi Biotec).

#### Effector-target coculture.

On day 11, purified CD4^+^ T cells from PLWH were infected in vitro with HIV-1. After 3 days of infection, cells were stained with 1 μM CellTrace Far Red (CTFR; Thermo Fisher Scientific) and cocultured with autologous CD8^+^ T cells at a 1:1 effector/target ratio in the absence of TB-PE.

For healthy donors, CD4^+^ T cells were pulsed with the CEF peptide pool for 1 hour at 37°C to generate antigen-specific target cells. These “target” cells were stained with 0.1 μM CTFR, whereas un-pulsed “bystander” CD4^+^ T cells were stained with 1 μM CTFR. Target, bystander, and expanded CD8^+^ T cells were cocultured at a 1:1:1 ratio (target/bystander/effector).

After 24 hours of coculture, CD8^+^ T cell–mediated killing was quantified by measurement of intracellular p24 expression by flow cytometry (82) in HIV-1–infected targets or by calculation of the ratio of bystander to target cells in CEF-pulsed assays.

### Fractionation of TB-PE

The polar and lipid fractions of TB-PE were isolated using the Bligh-Dyer protocol as previously detailed (57).

### Neutral lipid visualization and quantification

Isolated CD8^+^ T cells from healthy donors were cultured in serum-free RF10. Cells were incubated for 24 hours in the presence or absence of 20% vol/vol of pooled TB-PE. After incubation, cells were stained with 1.9 μM BODIPY 493/503 (Ambeed) fixed with 4% paraformaldehyde and adhered to poly-l-lysine–coated (Sigma-Aldrich) slides using the Statspin Cytofuge 2 (Beckman Coulter). Slides were stained with DAPI (Roche), and regions of interest were imaged at ×40 and ×100 using the Olympus VS200 slide scanner. Data were analyzed using cell detection and mean cell fluorescence measurements in QuPath (83).

### Statistics

Experimental data were analyzed using Prism (GraphPad Software). Normality of the data was tested using the Kolmogorov-Smirnov test or the Shapiro-Wilk test in a sample size–dependent manner. Based on the results of the relevant normality test, statistical significance was tested with a 2-tailed paired or unpaired *t* test, or a Wilcoxon’s signed-rank test to compare 2 conditions; a 1-way ANOVA followed by Tukey’s honestly significant difference (HSD) post-test or Kruskal-Wallis test followed by Dunn’s post-test was used for multiple comparisons. A *P* value less than 0.05 was considered significant. HIV-1 infection frequency per million cells was calculated using a binomial model (84).

### Study approval

This research was carried out in accordance with the Declaration of Helsinki (85) and was approved by the institutional review boards of the Hospital F.J. Muñiz (Buenos Aires, Argentina; approval PRIISA.BA–5334) and the Western Sydney Local Health District (2022/PID00100 – 2022/ETH00092). Buffy coats from healthy donors were obtained from the Australian Red Cross Blood Service, Sydney, Australia, and from Établissement Français du Sang, Toulouse, France, under contract 21/PLER/TOU/IPBS01/20,130,042. Written informed consent was obtained from all participants.

### Data availability

The GenBank accession numbers for sequences reported here are PX486032–PX486070, PX486071–PX486108, PX486109–PX486157, PX486158–PX486208, PX486209–PX486243, PX486244–PX486283, PX486284–PX486300, PX486301–PX486339, PX486340–PX486367, PX486368–PX486410, PX486411–PX486520, PX486521–PX486588, PX486589–PX486632, PX486633–PX486695, and PX486696–PX486729. scRNA-seq data are available in the NCBI’s Gene Expression Omnibus database under the accession number GSE312398. Raw data can be obtained from the Supporting Data Values spreadsheet. In-house scripts used for data analysis are available upon request.

## Author contributions

GD conceptualized and designed the study. GD and SC designed and conducted the majority of ex vivo and in vitro experiments. GD, SP, LB, and CV provided supervision, critical input, and funding support for the project. LB, MFQ, ADK, and GT provided clinical samples. JS and SC performed the transcriptomic analyses. JB and MM assisted with sample collection. APC, KF, and EL assisted with HIV-1 sequencing. FWVD and KB performed high-parameter flow cytometry. YL and TRO contributed to flow cytometry data analysis. DB, HR, and SC performed neutral lipid visualization. NN and ANH provided resources for flow cytometry and microscopy. JMR assisted with in vitro experiments. ZV performed metabolic assays. SC and GD wrote the original manuscript. LB and SP supervised and revised the final version of the manuscript. All authors reviewed the final version of the manuscript.

## Conflict of interest

The authors have declared that no conflict of interest exists.

## Funding support

This work is the result of NIH funding, in whole or in part, and is subject to the NIH Public Access Policy. Through acceptance of this federal funding, the NIH has been given a right to make the work publicly available through PubMed Central.

- Delaney AIDS Research Enterprise to Find a Cure (1UM1AI126611-01 and 1UM1AI164560-01 to SP).
- Australian National Health and Medical Research Council (APP1149990 to SP).
- amfAR, The Foundation for AIDS Research, Mathilde Krim Fellowship in Biomedical Research (110561-75-RKRL to GD).
- Argentinean National Agency of Promotion of Science and Technology (PICT 2019-2019-01044 and 46 PICT 2020-00501 to LB).
- Sidaction to CV.
- Sandra and David Ansley.
- GD’s salary support from the Sydney Medical School Foundation and The University of Sydney Institute for Infectious Diseases.
- A postdoctoral fellowship from the Agence Nationale de Recherche sur le Sida et les hépatites virales (ANRS I MIE) to ZV.
- SC, DB, FWVD, and YL by the Commonwealth of Australia through an Australian Government Research Training Program Scholarship.

## Supplementary Material

Supplemental data

Supporting data values

## Figures and Tables

**Figure 1:**
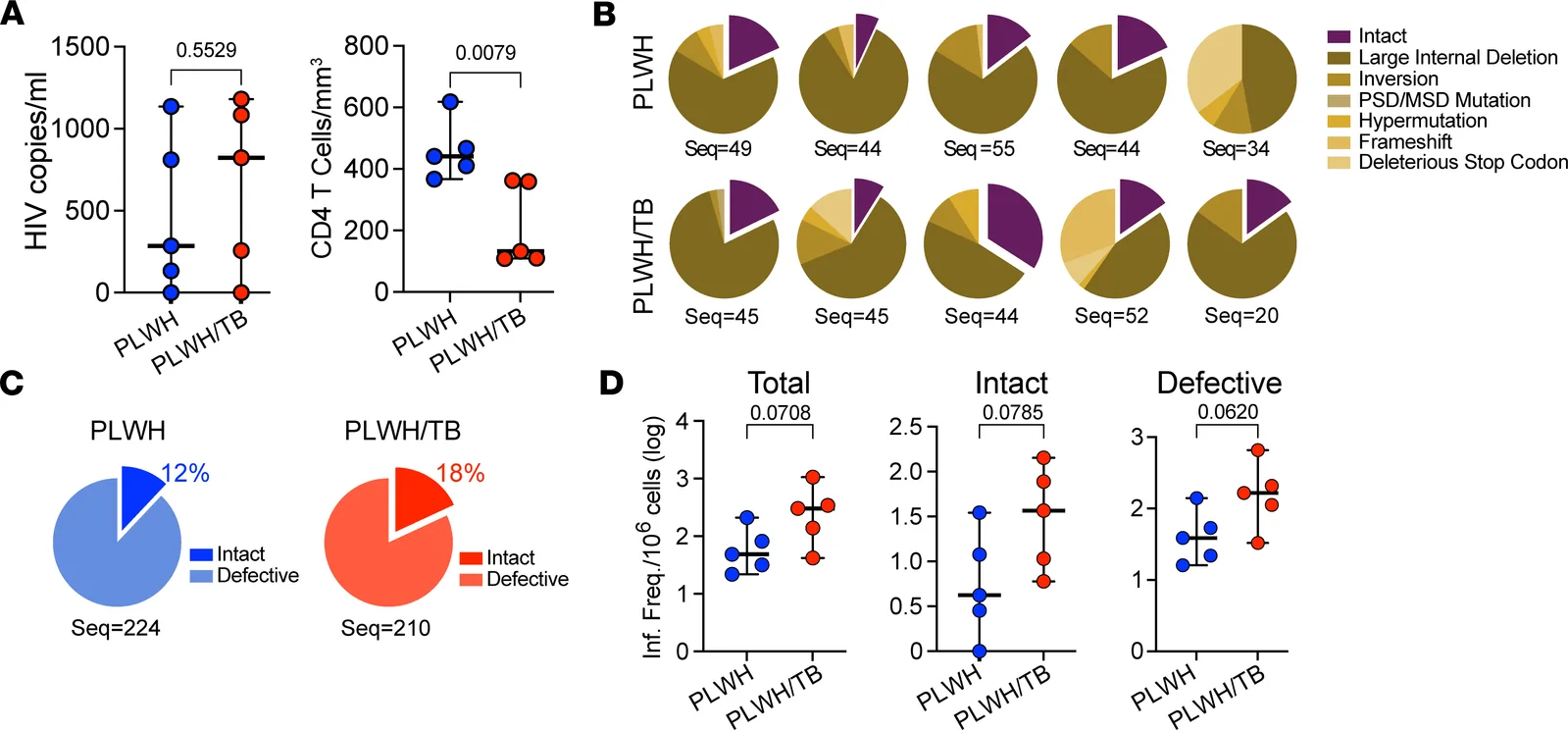
HIV-1 proviral genetic landscape in PLWH/TB. (**A**) Plasma HIV-1 RNA copies/mL and CD4^+^ T cell counts in 5 PLWH (blue) and 5 PLWH/TB (red). (**B**) Pie charts showing the proportion of genetically intact and defective proviral HIV-1 sequences for each donor. PSD, packaging signal deletion; MSD, major splice donor. (**C**) Averaged proportion of genetically intact and defective proviral HIV-1 sequences across groups. The total number of sequences analyzed per group is shown below each pie chart. (**D**) Infection frequency of total, genetically intact, and genetically defective proviruses per 10^6^ CD4^+^ T cells. Frequencies were calculated based on the proportion of CD4^+^ T cells in each sample and log-transformed. Each data point represents a single donor. Bars represent the median with 95% CI. Statistical significance was determined by 2-tailed paired *t* test, with *P* < 0.05 considered significant.

**Figure 2:**
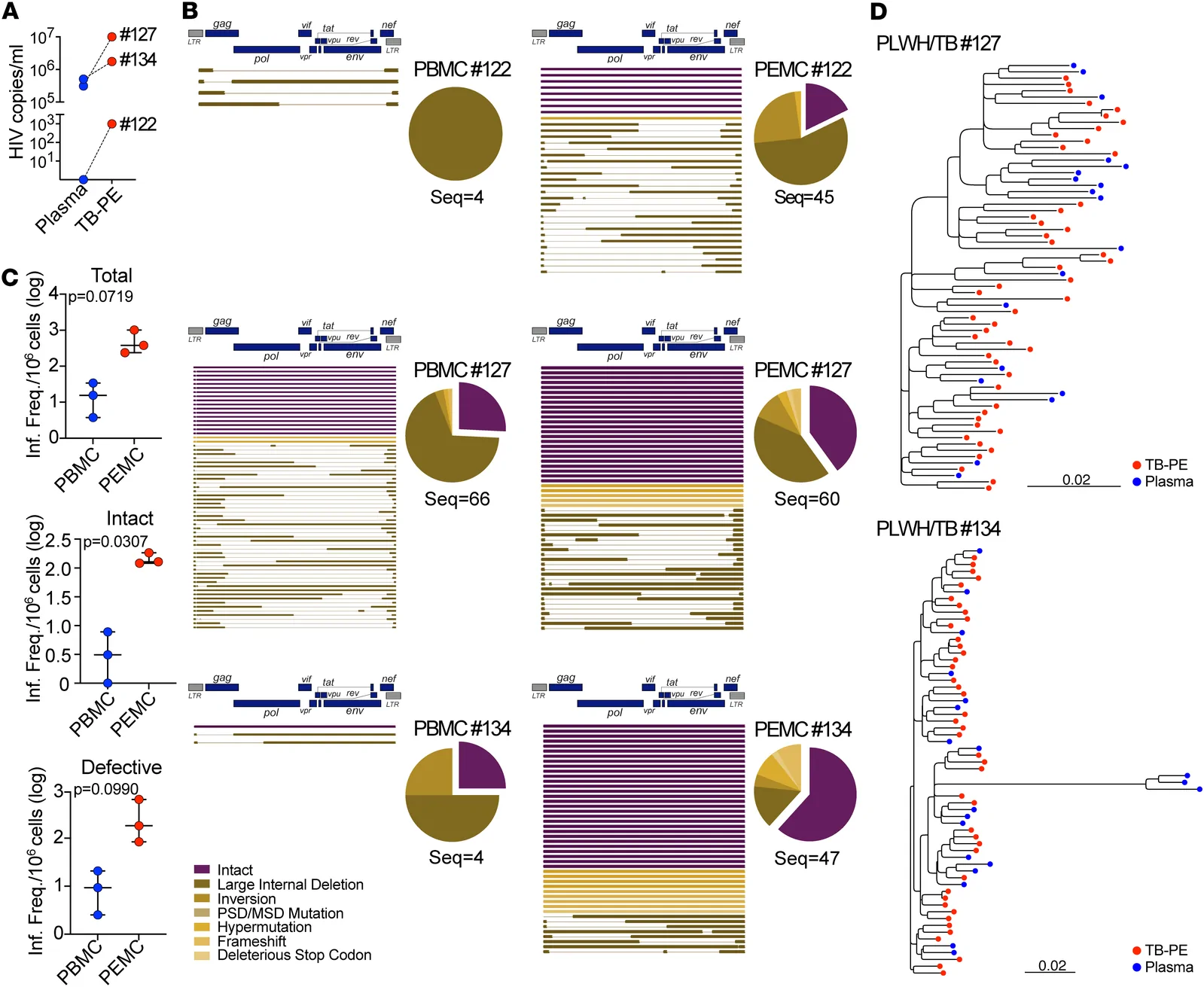
The site of HIV-1/*Mtb* coinfection is enriched in HIV-1 DNA and RNA. (**A**) HIV-1 RNA copies/mL in plasma (blue) and TB-PE (red) from 3 PLWH/TB. (**B**) Pie charts showing the proportion of genetically intact and defective proviral HIV-1 sequences derived from PBMCs and PEMCs for each donor. Horizontal lines adjacent to pie charts represent individual contig sequences aligned to HXB2. (**C**) Log-transformed infection frequency of total, genetically intact, and genetically defective proviruses per million cells. Each data point represents a single donor. Bars represent the median with 95% CI. Statistical significance was determined by 2-tailed paired *t* test, with *P* < 0.05 considered significant. (**D**) Phylogenetic tree of HIV-1 RNA sequences derived from plasma (blue) and TB-PE (red).

**Figure 3:**
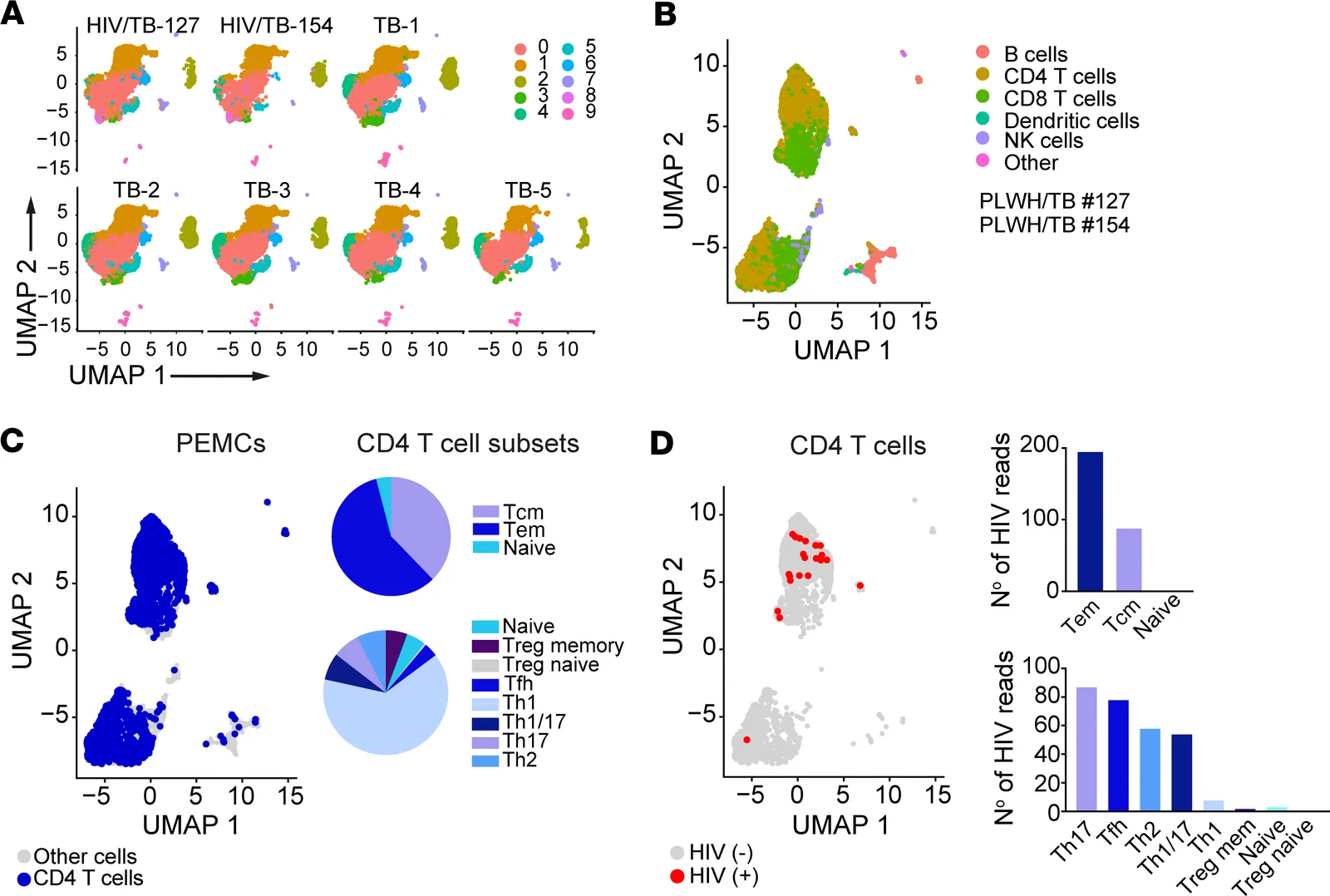
scRNA-seq of TB-PE reveals preferential infection of effector memory CD4^+^ T cells. (**A**) UMAP of PEMCs from 2 PLWH/TB analyzed in-house compared with PEMCs from 5 TB patients reported by Cai et al. (32). (**B**) UMAP of immune cell populations from integrated PEMCs. (**C**) UMAP showing clusters of CD4^+^ T cells within integrated PEMCs. Pie charts indicate the proportions of central memory (Tcm), effector memory (Tem), and naive CD4^+^ T cells (top) and naive, regulatory T cell (Treg) memory, Treg naive, and T helper (Th) subsets (bottom) across both donors. (**D**) UMAP highlighting cells expressing HIV-1 transcripts (red). Bar graphs show the distribution of HIV-1 reads among CD4^+^ T cell subsets. Tfh, follicular helper T cells.

**Figure 4:**
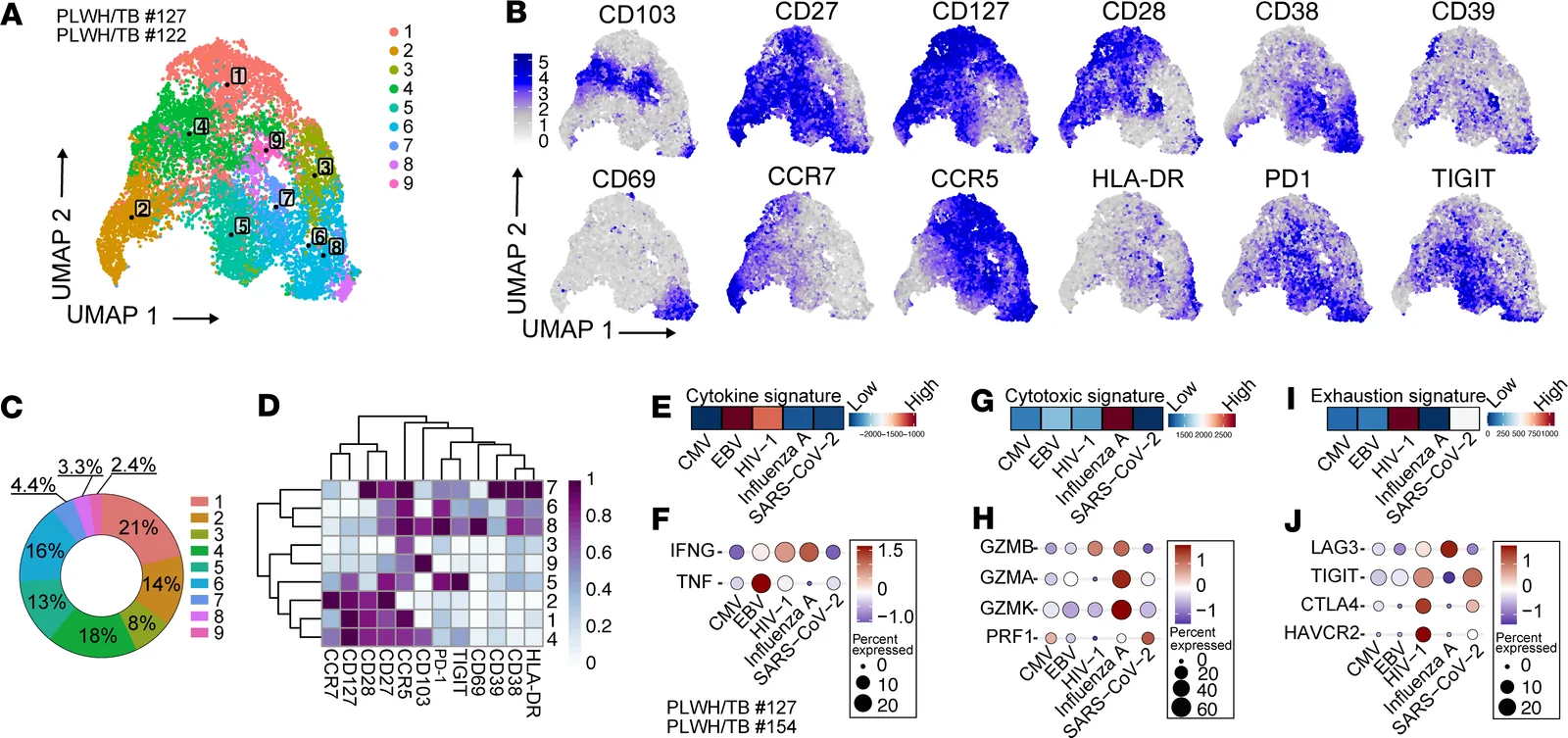
Immunophenotype of TB-PE–derived CD8^+^ T cells. (**A**) UMAP showing unbiased clustering of CD8^+^ T cells from 2 PLWH/TB based on the immune markers CD103, CD69, CD27, CCR7, CD127, CCR5, CD28, HLA-DR, CD38, PD-1, CD39, and TIGIT. (**B**) UMAP of expression levels of selected immune markers in TB-PE–derived CD8^+^ T cells from both donors. (**C**) Proportions of CD8^+^ T cell clusters identified by UMAP analysis. (**D**) Heatmap of average expression of 12 immune markers across clusters. (**E**–**J**) scRNA/TCR-seq analysis of TB-PE–derived virus-specific CD8^+^ T cells from 2 PLWH/TB. (**E**) Cytokine signature analysis. (**F**) Dot plot showing relative expression of IFNG and TNF. (**G**) Cytotoxicity signature analysis. (**H**) Dot plot showing relative expression of GZMA, GZMB, GZMK, and PRF1. (**I**) Exhaustion signature analysis. (**J**) Dot plot showing relative expression of TIGIT, LAG3, CTLA4, and HAVCR2.

**Figure 5:**
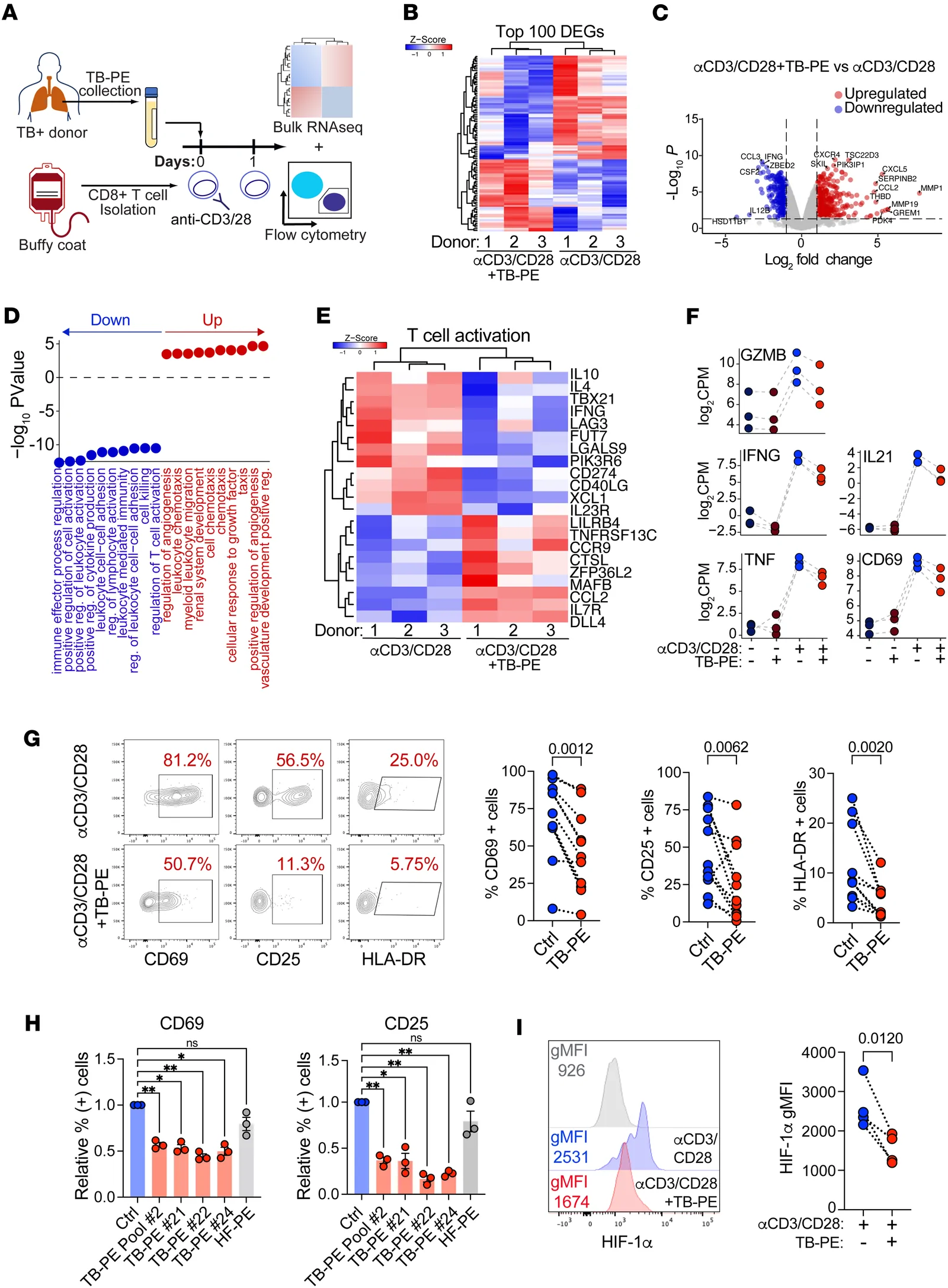
TB-PE treatment downmodulates T cell effector functionality. (**A**) Schematic of the experimental model used to study the effect of TB-PE on CD8^+^ T cell functionality. (**B**) Heatmap showing hierarchical clustering of the top 100 variable genes between TB-PE–treated and control CD8^+^ T cells stimulated with anti-CD3/CD28 antibodies. (**C**) Volcano plot of differentially expressed genes (DEGs). (**D**) Gene set enrichment analysis (GSEA) of DEGs (*P* < 0.05 and absolute log fold change > 1) comparing TB-PE–treated and control activated CD8^+^ T cells. Dot plots illustrate the top 10 upregulated and downmodulated enriched Gene Ontology biological process terms. (**E**) Heatmap of normalized expression of selected genes involved in T cell activation. (**F**) Gene expression levels of GZMB, IFNG, TNF, IL21, and CD69 across conditions. (**G**) CD8^+^ T cells were stimulated with anti-CD3/CD28 antibodies in the presence of TB-PE; cells activated in the absence of PE served as controls. Expression of the activation markers CD69, CD25, and HLA-DR was measured by flow cytometry after 24 hours (CD69, CD25) or 48 hours (HLA-DR). Representative flow plots (left) and quantification of activated cells (right) are shown. (**H**) Proportions of CD69^+^ (left) or CD25^+^ (right) cells stimulated in the presence of an additional TB-PE pool, 3 independent TB-PE samples (red), or HF-PE (gray). Values are relative to the non-PE control condition (blue). Data are shown as mean ± SD. Each dot represents an independent donor. Statistical significance was determined by paired 2-tailed *t* test or 1-way ANOVA followed by Tukey’s honestly significant difference (HSD) post-test. *P ≤ 0.05, **P ≤ 0.01. (**I**) HIF-1α expression in activated CD8^+^ T cells determined by flow cytometry. gMFI, geometric mean fluorescence intensity. Each dot represents an independent donor. Statistical significance was determined by paired 2-tailed *t* test or 1-way ANOVA followed by Tukey’s HSD post-test. *P ≤ 0.05, **P ≤ 0.01.

**Figure 6:**
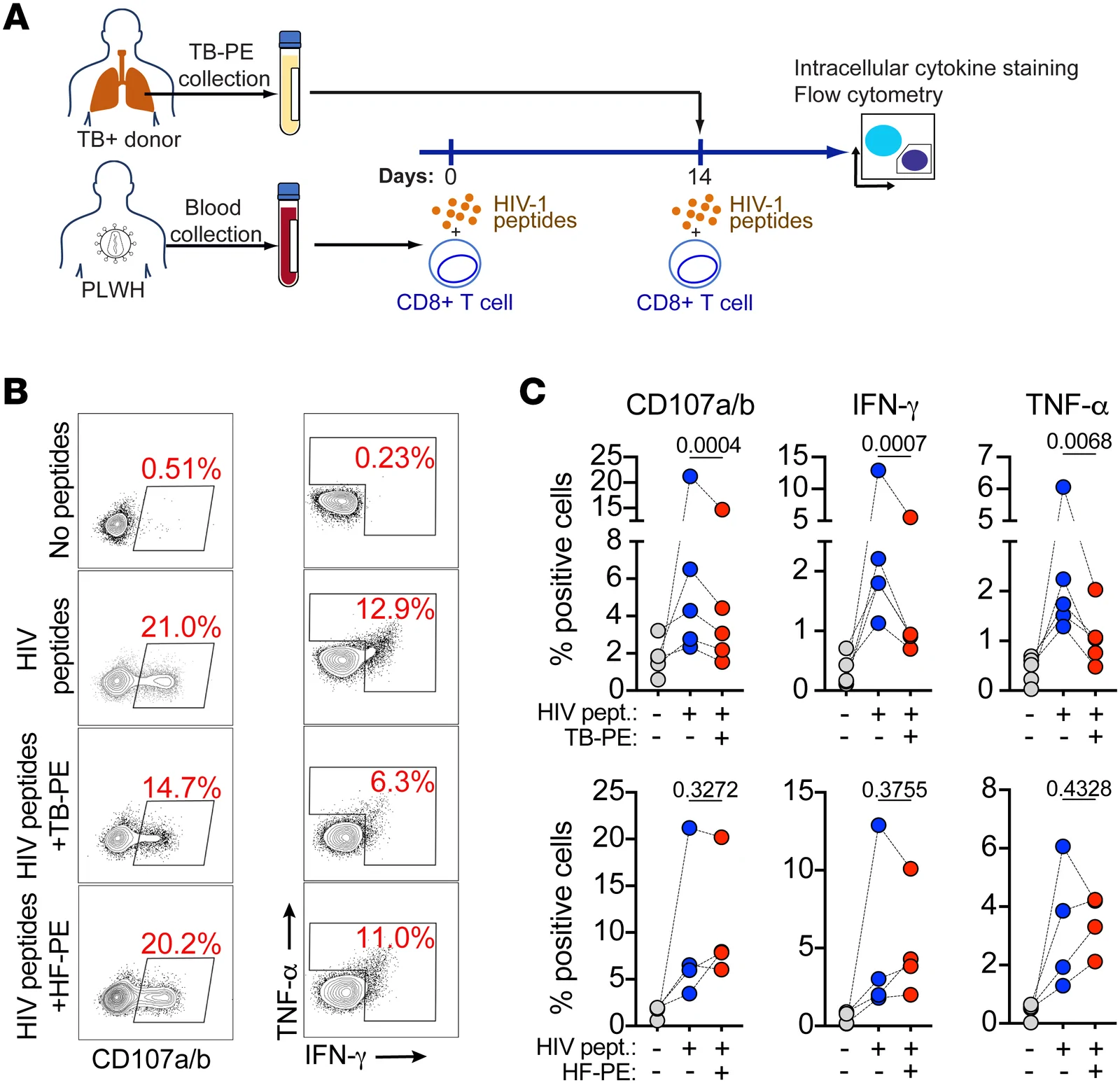
The effector anti–HIV-1 responses of CD8^+^ T cells are inhibited by TB-PE. (**A**) Schematic of the experimental model. (**B**) Representative flow cytometry of PLWH-derived CD8^+^ T cells expressing CD107a/b, TNF-α, and IFN-γ as markers for effector response upon restimulation with HIV-1 peptides in the presence or absence of TB-PE. Cells exposed to no peptides were used as an experimental control. Cells exposed to HF-PE were used as a control to determine the specific effect of TB-PE. (**C**) Quantification of expression of CD107a/b, TNF-α, and IFN-γ in PLWH-derived CD8^+^ T cells exposed to TB-PE (*n* = 5; top) or HF-PE (*n* = 4; bottom). Each data point represents a single donor. Statistical significance was determined by 2-tailed paired *t* test, with *P* < 0.05 considered significant.

**Figure 7:**
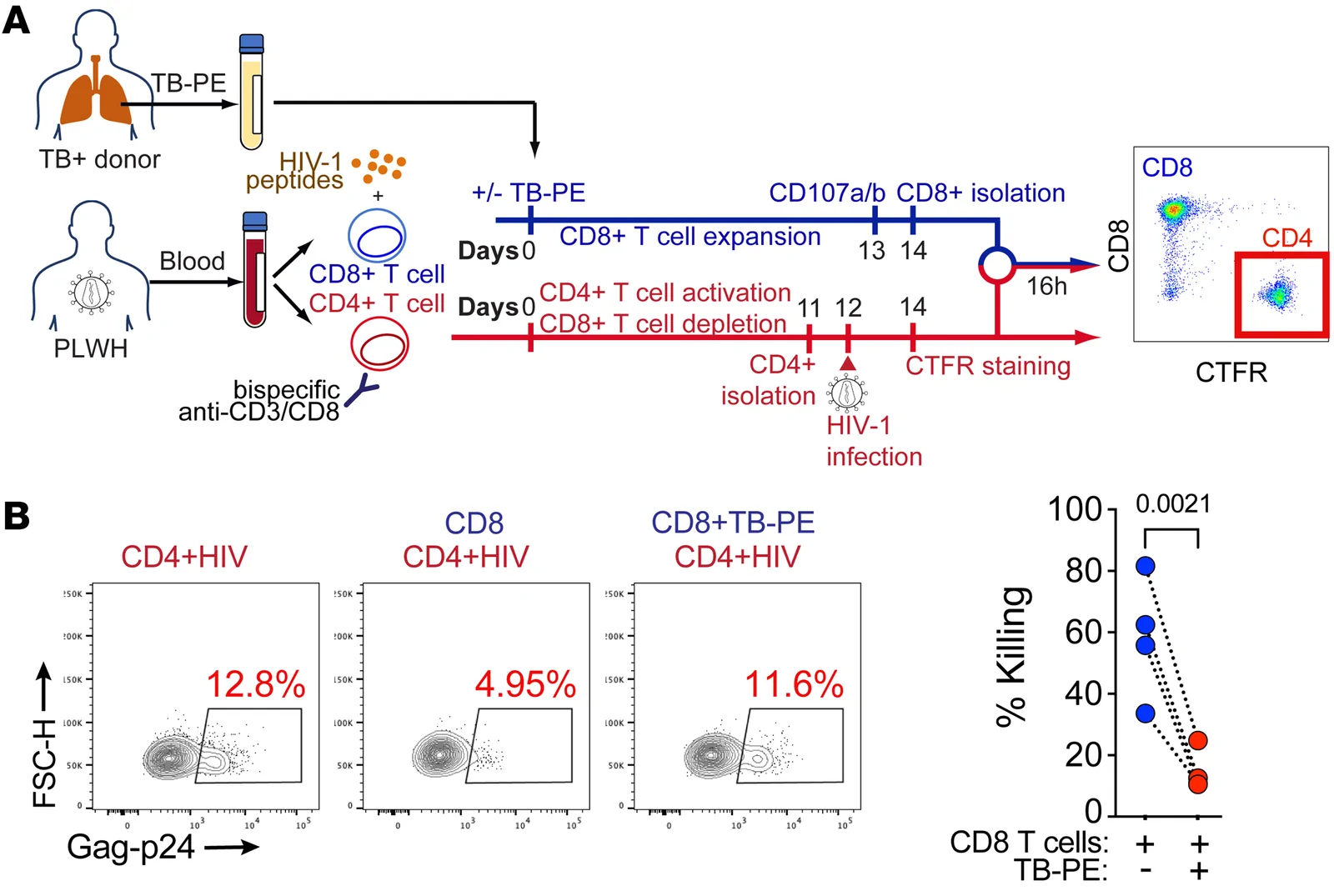
TB-PE inhibits the cytotoxic function of HIV-1–specific CD8^+^ T cells. (**A**) Schematic of the experimental model. (**B**) Representative flow cytometry plots showing p24 levels in HIV-1–infected CD4^+^ T cells cocultured with CD8^+^ T cells expanded in the presence or absence of TB-PE. As a control, CD4^+^ T cells were cultured without CD8^+^ T cells. Quantification of HIV-1–infected cell killing was performed using samples from 4 PLWH. Each data point represents a single donor. Statistical significance was determined by 2-tailed paired *t* test, with *P* < 0.05 considered significant.

**Figure 8:**
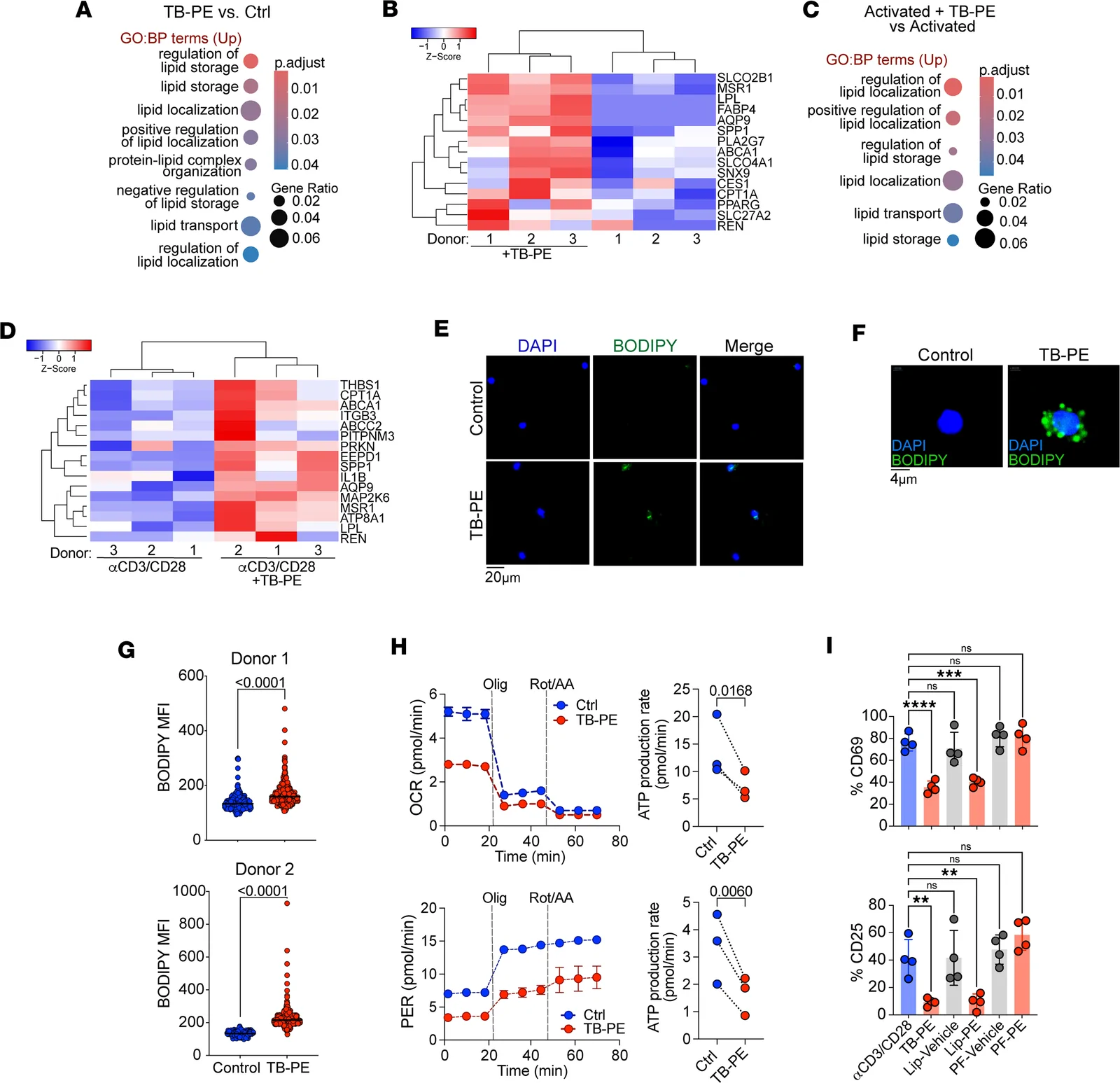
TB-PE–derived lipids accumulate in CD8^+^ T cells and inhibit T cell activation. (**A**) Dot plot illustrating lipid-associated pathways upregulated in TB-PE–exposed resting CD8^+^ T cells. (**B**) Heatmap presenting normalized expression values of lipid-associated genes between unstimulated control and TB-PE–exposed cells. (**C**) Dot plot illustrating upregulated lipid-associated pathways in CD8^+^ T cells stimulated with anti-CD3/CD28 antibodies exposed to TB-PE when compared with control CD8^+^ T cells. (**D**) Heatmap of normalized expression values of lipid-associated genes between anti-CD3/CD28–treated control and TB-PE–exposed CD8^+^ T cells. (**E** and **F**) Representative images at ×40 (**E**) and ×100 (**F**) original magnification of neutral lipid staining in CD8^+^ T cells in the presence and absence of TB-PE. Scale bars: 20 μm (**E**), 4 μm (**F**). (**G**) Quantification of BODIPY mean fluorescence intensity (MFI) between control and TB-PE–exposed CD8^+^ T cells. (**H**) Top: Oxidative phosphorylation measured as oxygen consumption rate (OCR; left) and mitochondria-dependent ATP production (right). Bottom: Glycolysis measured as proton efflux rate (PER; left) and glycolysis-dependent ATP production (right). Olig, oligomycin; Rot, rotenone; AA, antimycin A. (**I**) Proportion of CD69^+^ (top) and CD25^+^ (bottom) cells stimulated with anti-CD3/CD28 antibodies in the presence of either pooled TB-PE (red), the lipid fraction (Lip-PE) (red), lipid vehicle (gray), the polar fraction (PF-PE) (red), or the polar fraction vehicle (gray). Data are shown as mean ± SD. Each dot represents an independent donor. Statistical significance was determined by paired 2-tailed *t* test or 1-way ANOVA followed by Tukey’s HSD post-test. **P ≤ 0.01, ***P ≤ 0.001, ****P ≤ 0.0001.

**Table 1:**
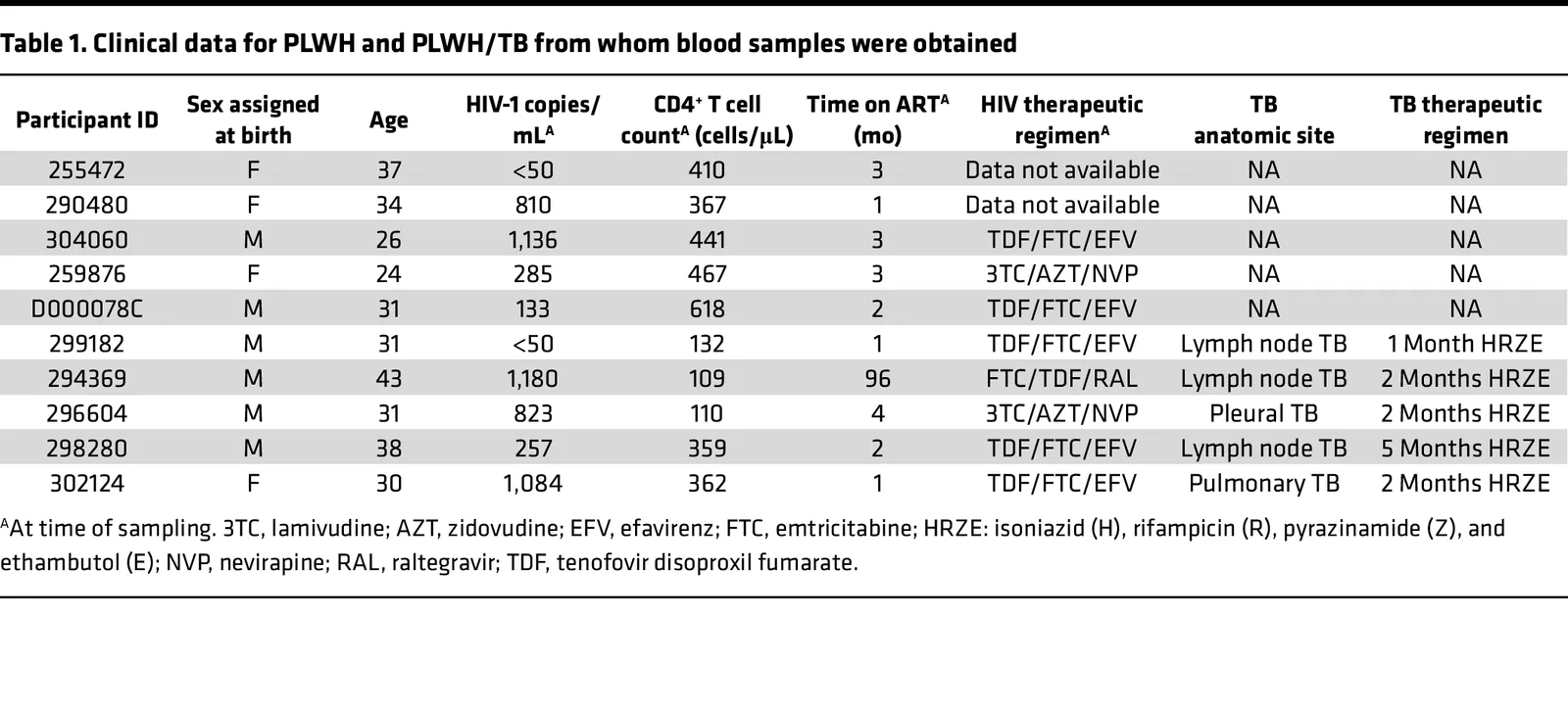
Clinical data for PLWH and PLWH/TB from whom blood samples were obtained

**Table 2:**
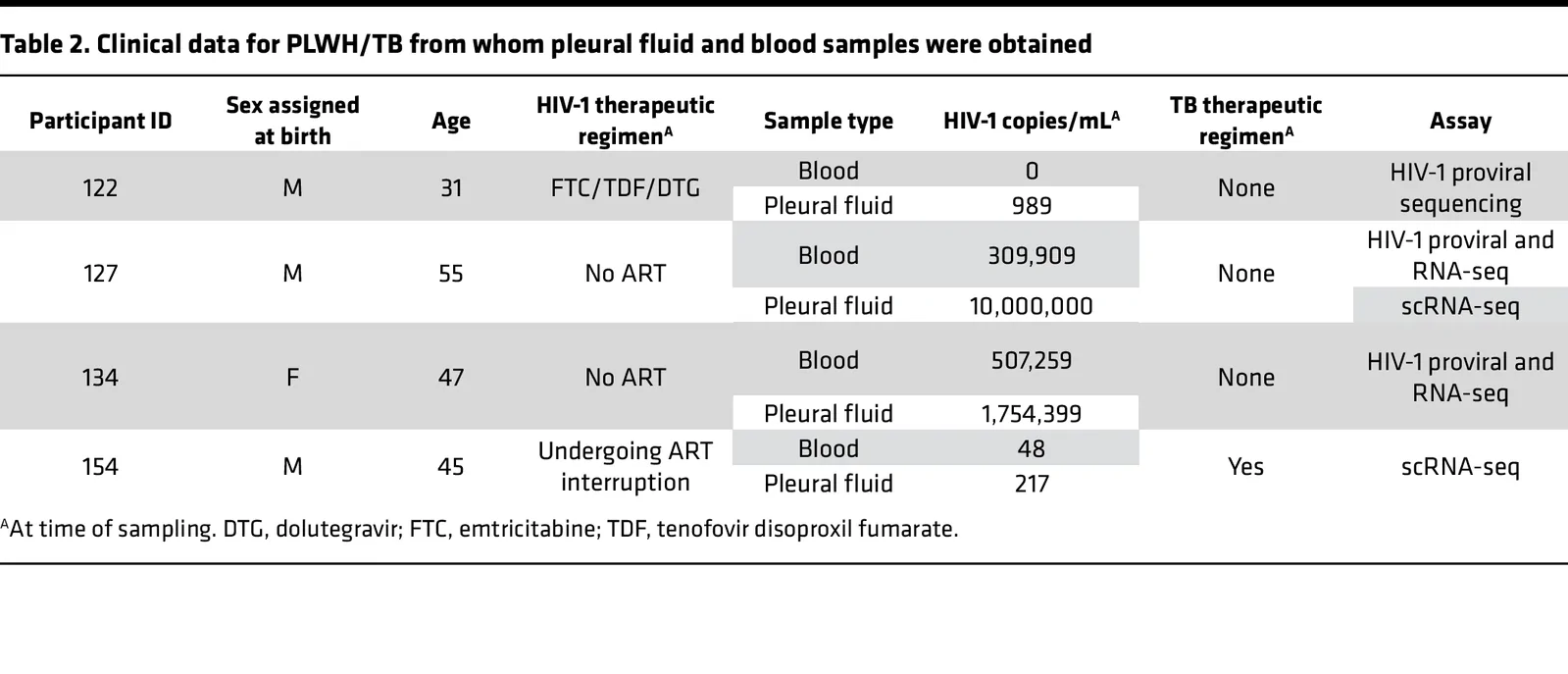
Clinical data for PLWH/TB from whom pleural fluid and blood samples were obtained

## Version 1 08/20/2026

In-Press Preview

## Version 2 10/08/2026

Electronic publication
