# Supplementary material for: The pleural tuberculosis–associated microenvironment promotes HIV-1 persistence by impairing CD8+ T cell–mediated viral control
Source: JCI Insight. 2026 Aug 20;11(19):e201268. doi: 10.1172/jci.insight.201268 (PMC13726412; doi:10.1172/jci.insight.201268)
Supplement: Supplementary file 1 [file jciinsight-11-201268-s053.pdf]

Supplemental Material

The pleural tuberculosis-associated microenvironment promotes HIV persistence by impairing CD8+ T-cell mediated viral control

Samantha Cronin, Jennifer Simpson, Andrea Pereyra-Casanova, Yuchen Li, Josefina Marín-Rojas, Freja Warner van Dijk, Katie Fisher, Daniel Buffa, Hafsa Rana, Zoï Vahlas, Joaquina Barros, Mariano Maio, Thomas R. O'Neil, Kirstie Bertram, Eunok Lee, Najla Nasr, Andrew N. Harman, Gabriela Turk, Maria F. Quiroga, Anthony D. Kelleher, Christel Vérollet, Luciana Balboa, Sarah Palmer, Gabriel Duette

Supplementary tables

Table S1. Participant Characteristics for HIV-negative donors with Pleural Effusion.

	**TB-PE**	**HF-PE**
**Age (years, range)**	35 (19-52)	50 (35-66)
**Gender, male %**	M, 78%	M, 80%
**Nationality, %**	Argentina, 56% Bolivia, 33% Peru, 11%	Argentina, 100%
**TB disease %**	Pleural, 22% Pleural and Pulmonary, 78%*	-
**Etiology**	*M. tuberculosis*	ND

*Confirmed by chest X-Ray

Table S2. Clinical data for PLWH included in this study

Participant ID	Age (years)	Sex	Time on therapy[A] (years)	Viral load[A] (copies/ml)	Antiretroviral regimen[A]
PS02017	29	Male	3	<50	NVP, TRU
PS02018	46	Male	3	<50	3TC, DDI, EFV, TRU
PS03022	20	Male	4	<50	ABC, 3TC
PS01008	29	Male	2	<50	SQV
PS01007	42	Male	4	<50	ABC, 3TC

3TC, lamivudine; ABC, abacavir; DDI, didanosine; EFV, efavirenz; NVP, nevirapine; TRU, truvada, SQV, saquinavir.

[A]At time of sampling.

Table S3. Antibodies Used for High-Parameter Flow Cytometry

Antibody Name	Clone	Source	Catalogue Number
CD28-BUV395	CD28.2	BD Biosciences	740308
CD8-BUV496	SK1	BD Biosciences	741199
HLADQ-BUV563	TU169	BD Biosciences	748563
CD161-BUV615	HP-3G10	BD Biosciences	751374
CD154-BUV737	TRAP1	BD Biosciences	748983
CD4-BUV805	OKT4	BD Biosciences	750976
CCR7-BV421	2-LI-A	BD Biosciences	562555
CD39-BV480	TU66	BD Biosciences	746454
HLADR-BV605	G46-6	BD Biosciences	562845
PD1-BV711	EH12.1	BD Biosciences	564017
CD27-BV786	LI27	BD Biosciences	563327
CCR5-VioB515	REA245	Miltenyi Biotec	130120022
CD3-NovaFluorB610	UCHT1	Thermo Fisher Scientific	H002T03B06-A
CD69-BB700	FN50	BD Biosciences	569399
TIGIT-PE	A15153G	BioLegend	372704
CD103-PEVio615	REA803	Miltenyi Biotec	130111837
CD127-PECY7	R34.34	Thermo Fisher Scientific	25-1271-82
CD38-APCFire810	HIT2	BioLegend	303550

Supplementary Figures

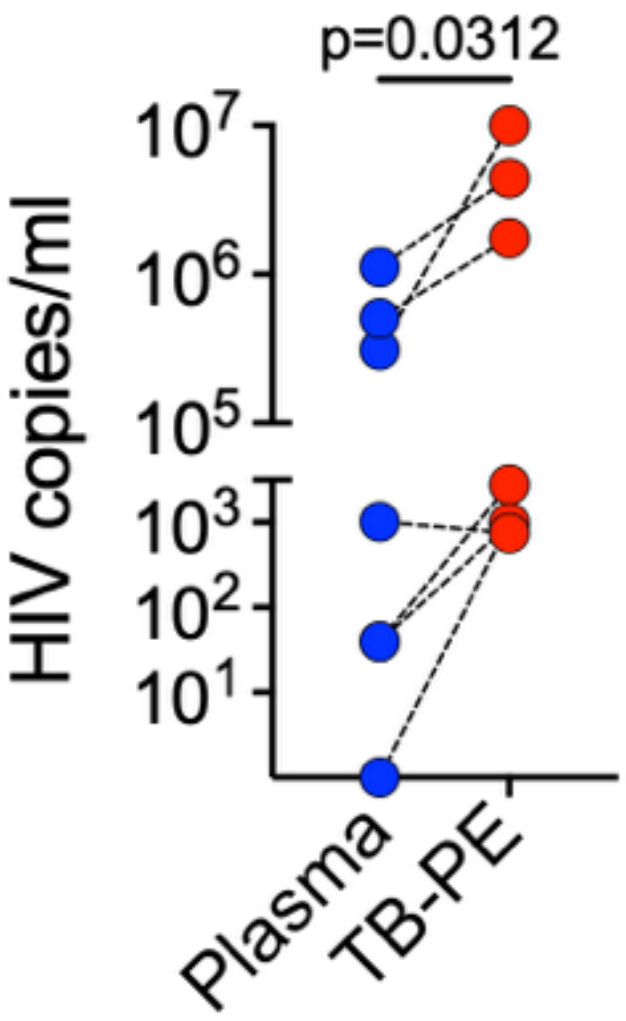

Supplementary Figure 1. HIV-1 viral load is higher in TB-PE. *HIV-1 RNA copies per ml in plasma (blue) and tuberculous pleural effusion (red) from seven people living with HIV-1 and pleural TB.*

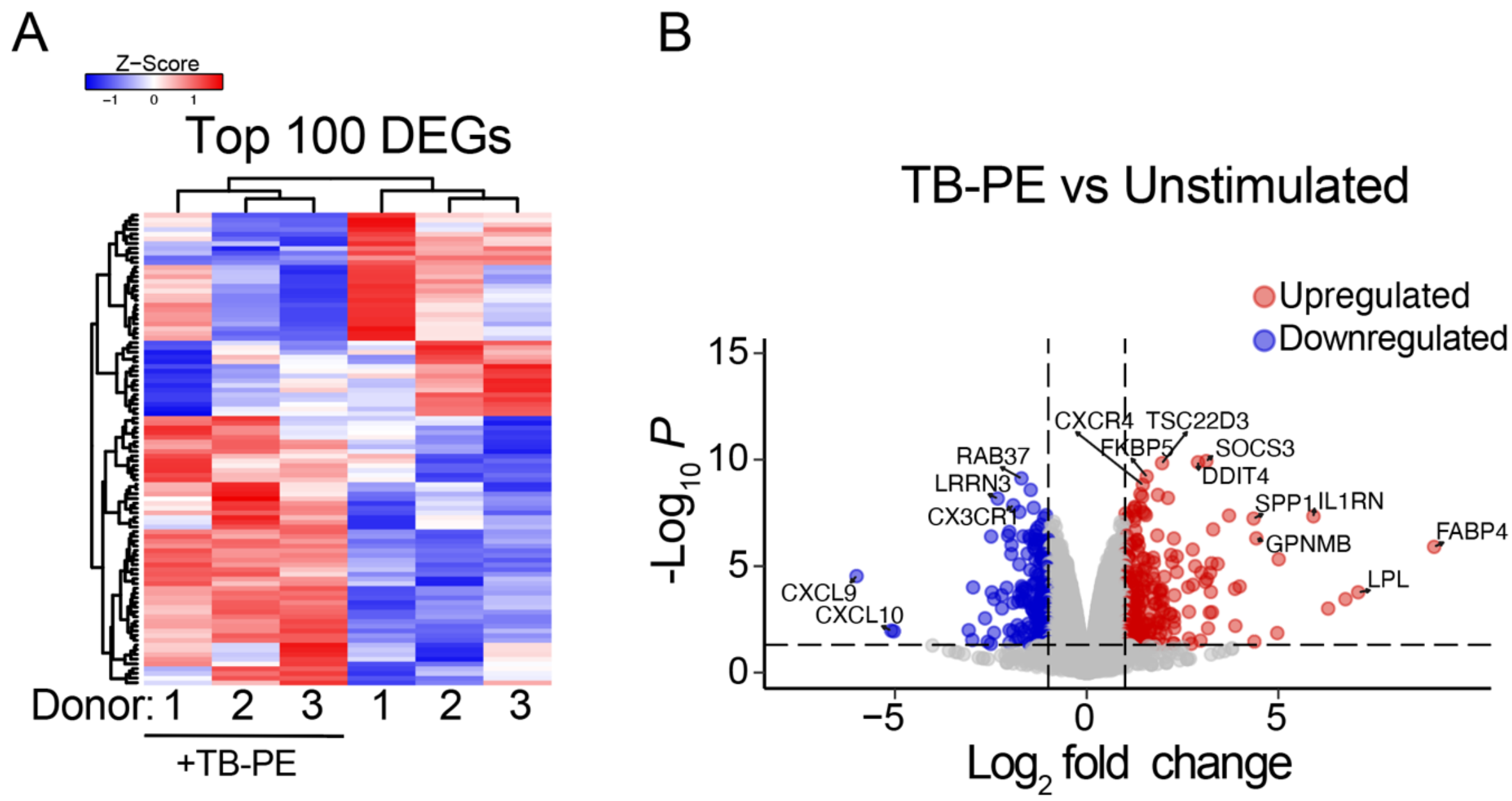

Supplementary Figure 2. TB-PE treatment alters the transcriptome of CD8+ T cells. *(A) Heatmap showing hierarchical clustering of the top 100 differentially expressed genes (DEGs) between TB-PE treated and control CD8+ T cells. (B) Volcano plot of differentially expressed genes with significantly upregulated genes in red and significantly downregulated genes in blue ($p<0.05$).*

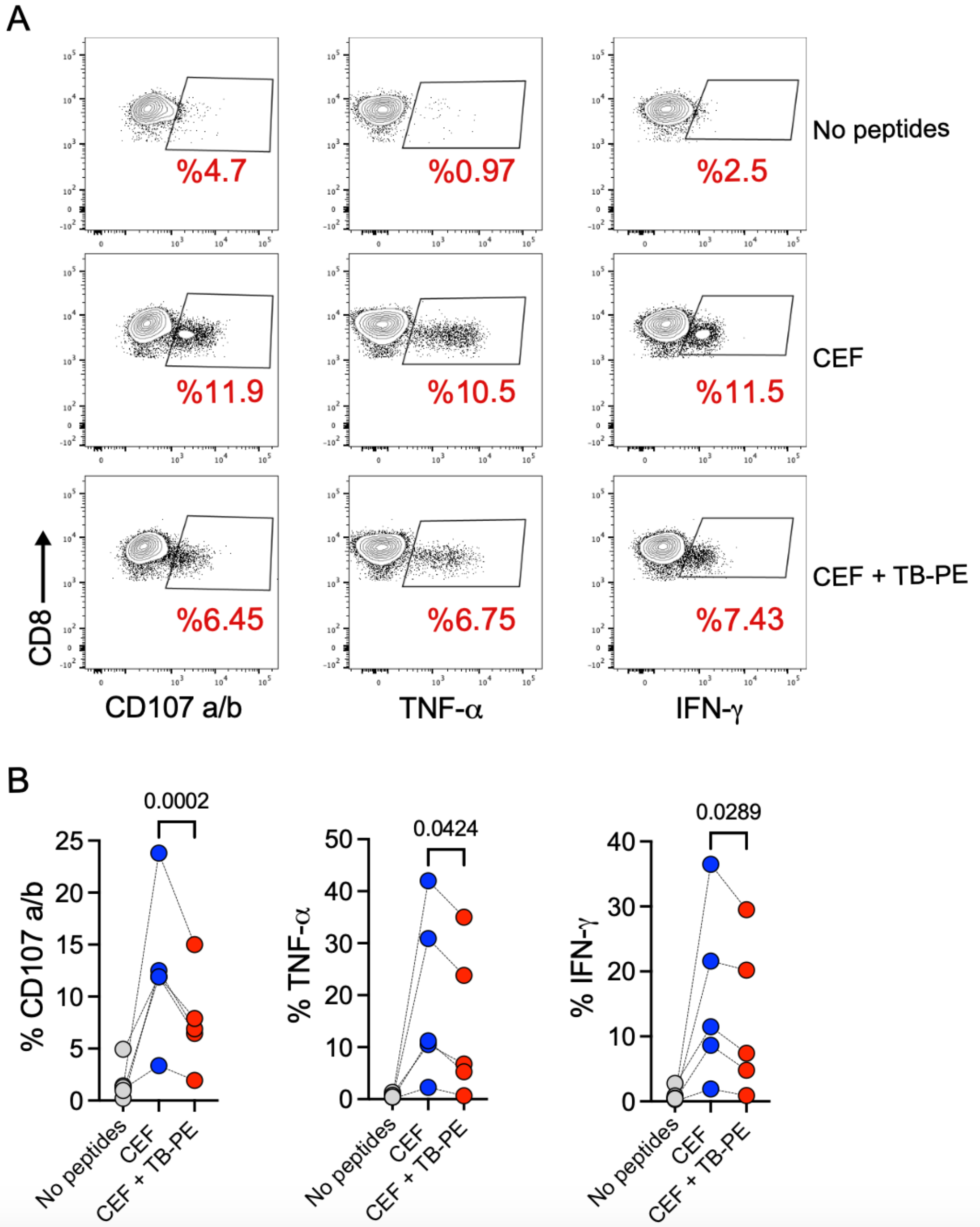

Supplementary Figure 3. The effector antiviral responses of CD8+ T cells are inhibited by TB-PE. *(A) Representative flow cytometry of healthy donor-derived CD8+ T cells expressing CD107a/b, TNF-α, and IFN-γ as markers of effector response upon restimulation with pooled peptides from cytomegalovirus, influenza, and Epstein-Barr virus (CEF peptide pool) in the presence (red) or absence (blue) of TB-PE. Cells exposed to no peptides (grey) were used as an experimental control. (B) Quantification of the expression of CD107a/b, TNF-α, and IFN-γ in healthy donor-derived CD8+ T cells exposed to TB-PE (n=5). Each data point represents one donor. Statistical significance was assessed by paired t-test with $p<0.05$ considered significant.*

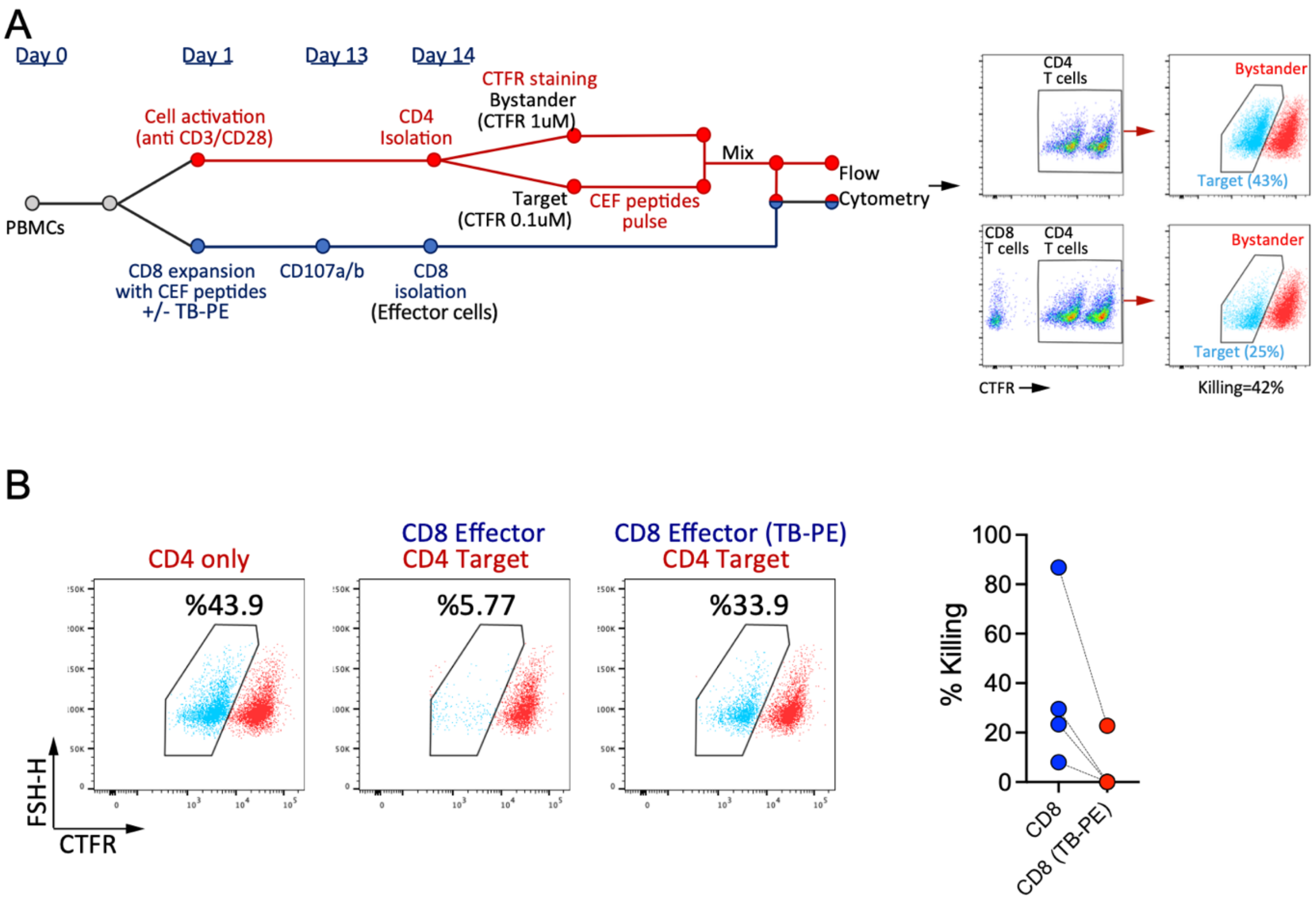

Supplementary Figure 4. TB-PE inhibits virus-specific CD8+ T cell cytotoxicity. *(A) Schematic of the experimental model used to study the effect of TB-PE on virus-specific CD8+ T cell cytotoxicity. CD8+ T cells were expanded with a pool of peptides derived from Cytomegalovirus, Epstein Barr Virus and Influenza (CEF peptide pool) in the presence/absence of TB-PE. (B) Representative flow cytometry plots showing the proportion of target cells after coculture with CEF-specific CD8+ T cells. As a control, CD4+ T cells were cultured without CD8+ T cells (left). Quantification of CD8+ T cell-mediated killing of cells pulsed with CEF peptides (right). Each dot represents a different donor.*
